# Online social cohesion reflects real-world group action in Syria during the Arab Spring

**DOI:** 10.1371/journal.pone.0254087

**Published:** 2021-07-16

**Authors:** Megan Chiovaro, Leah C. Windsor, Alistair Windsor, Alexandra Paxton

**Affiliations:** 1 Department of Psychological Sciences, University of Connecticut, Mansfield, Connecticut, United States of America; 2 Center for the Ecological Study of Perception and Action, University of Connecticut, Mansfield, Connecticut, United States of America; 3 Institute for Intelligent Systems, University of Memphis, Memphis, Tennessee, United States of America; 4 Department of Mathematical Sciences, University of Memphis, Memphis, Tennessee, United States of America; Universidade Estadual de Maringa, BRAZIL

## Abstract

In recent years, political activists have taken to social media platforms to rapidly reach broad audiences. Despite the prevalence of micro-blogging in these sociopolitical movements, the degree to which virtual mobilization reflects or drives real-world movements is unclear. Here, we explore the dynamics of real-world events and Twitter social cohesion in Syria during the Arab Spring. Using the nonlinear methods cross-recurrence quantification analysis and windowed cross-recurrence quantification analysis, we investigate if frequency of events of different intensities are coupled with social cohesion found in Syrian tweets. Results indicate that online social cohesion is coupled with the counts of all, positive, and negative events each day but shows a decreased connection to negative events when outwardly directed events (i.e., source events) were considered. We conclude with a discussion of implications and applications of nonlinear methods in political science research.

## Introduction

Before social media, people coordinated protests using phone tree calling sheets and mimeographed flyers, through in-person meetings of social organizations and churches, and by word of mouth. They gathered together and hand-made protest signs that reflected their agendas. As McAdam found, people were more likely to join movements when they had a personal connection [1, 2]: Historically, strong ties have been strong predictors of involvement in high-risk protest activity.

The cohesiveness and strength of social movements before the Internet were operationalized by the number of people present and through the political events that precipitated and resulted from social gatherings *en masse*. Cohesive social movements were reflected in their cohesive placards and posters. For example, the pervasive “I am a man” posters during the 1968 Memphis sanitation workers’ strike reflected their internal cohesion and unified, goal-oriented movement [3].

However, the rise of the Internet and its decentralized online networks have upended theories about recruitment to—and participation in—social movements. Strong interpersonal ties have yielded to weak impersonal ties that strongly predict involvement in social movements [4]. Measurements of social movements have similarly evolved from ethnographic interviews to “big data” analyses of social media.

Amid the cacophony of messages during political upheaval—such as the Arab Spring of the early 2010s—consistent themes emerge through hashtags, retweets, and the repeated mentions of concepts such as regime, demonstration, and human rights. The cohesiveness of these themes (i.e., the degree to which participants in the movement are metaphorically singing in one voice) reflects the real-world events happening contemporaneously with collective action in social movements or civil conflicts.

In the present research, we investigate the relationship between linguistic cohesion and real-world action in times of social conflict and unrest. We tackle a question related to the one posed by Zeitzoff [5]: How does social media reflect on-the-ground realities during times of social conflict? To do so, we examine patterns of linguistic cohesion on Twitter and their connection to real-world events during the Arab Spring uprising in Syria between March and June 2012.

Disentangling the complicated relationship between mass online mobilization—and social mobilization in person—continues to be relevant, even as our modes of communication change. Accordingly, the political events around the Arab Spring are still being understood. As Zeitzoff [5] recognizes, social media is changing conflict, but the mechanisms and relationships by which it does so are still largely unknown.

### Syria in context

The decades preceding the Arab Spring in Syria were marked by repression and fear. In 1971, Hafez al-Assad overthrew the previous leader and remained in power for thirty years, presiding during regional contentiousness and cooperation until his death in 2000. His son, Bashar al-Assad, continued the regime of authoritarian leadership after assuming his office. The Assad lineage hails from a minority Islamic sect (the Alawites) in a multi-religious and multi-ethnic country comprising Christians, Sunni and Shia Muslims, Kurds, and Palestinian refugees.

In December 2010, a Tunisian man named Mohamed Bouazizi died after setting himself on fire to protest the authoritarian government’s unjustifiable confiscation of his vendor cart. His actions served as a catalyst for the Tunisian Revolution, which rapidly spread to surrounding countries also subject to unjust authoritarian rule, including Egypt, Libya, and Syria.

Following a wave of democratization efforts across the Middle East and North Africa, modest Syrian protests began in March 2011. The regime responded harshly with imprisonments, detentions, censorship, and military operations as the resistance to Assad’s regime spread. The fighting escalated dramatically in March 2012, after increased unrest in the western city of Homs. International actors—including the Red Cross/Red Crescent Society, the United Nations General Assembly, the U.N. Security Council, and individual world leaders—worked toward cease-fires, negotiations, and concessions without much success. While many high-level military and diplomatic officials defected, numerous loyal forces continued the regime’s repression, and the Syrian people turned to social media to coordinate their activism and communicate to the world. Between March and June 2012, millions of tweets were sent within Syria, revealing the details of the conflict suppressed by the government. A full account of the history of the Arab Spring is outside of the scope of the current article; more information can be found in [6] and [7].

Syria is a multi-ethnic, multi-lingual, and multi-religious state. Kurdish people make up between 10–15% of the population, Alawites between 8–15%, and Christians about 10%. There are also much smaller sects (e.g., Ismailis, Druze), but Sunni Muslims comprise the vast majority of society. Most people speak Arabic as their primary language. Most policy reports addressing the current conflict report on the cohesion of the ruling Alawite military elite, rather than on the cohesiveness of the opposition movement. However, the Sunni majority’s disaffection with the Syrian government and their lack of political representation provide sufficient grievance and collective group identification to make them a cohesive force.

### Social media and social movements

Massive online social cohesion can manifest in real-world social behavior. Social media platforms enable online social cohesion by offering distributed information-gathering and real-time information dissemination. Given its accessibility and proficiency as an information exchange tool, Twitter has provided social and political activists an opportunity to complement [8], and not hinder participation in [9, 10], real-world social mobilization. During the 2012 Italian protests for global economic change, tweeting was determined to be more effective locally in discussing real-world, real-time events than traditional mainstream media platforms [11]. As people increasingly rely on social media for local news and information, we must understand exactly how these micro-blogs connect to and reflect real-world action.

Along with Facebook, Twitter has been credited as essential to the Arab Spring—both for protesters and for the government’s response to them [12]. The Arab Spring has been deeply examined within the political science literature [13, 14], but to our knowledge, the direct *coupling* between Syrian Twitter cohesion and international action has not been investigated (though hashtag usage has been linked to next-day protests; [15]). In fact, some have been skeptical about the effect of social media on collective action at all [16].

This skepticism is summarized in a quote by Lisa Anderson [17]:

The important story about the 2011 Arab revolts in Tunisia, Egypt, and Libya is not how the globalization of the norms of civic engagement shaped the protesters’ aspirations. Nor is it about how activists used technology to share ideas and tactics. Instead, the critical issue is how and why these ambitions and techniques resonated in their various local contexts. (p. 2)

Others have similarly expressed hesitation about the role of online organizing. Some have argued that real-life strong ties outweigh online weak ties and that the power of the strong ties through in-person contact is critical to recruiting people for higher-risk activities [1, 18].

Interestingly, these views may help make the case for bridging social media and social mobilization. Such views suggest the strength of online social ties is inextricably linked to the *context* and *ambitions* of the actors. Moreover, research suggests that weak ties still retain considerable political power: While strong ties tend to bind smaller groups, weak ties tend to bind groups across distances [19].

Social media generates critical information about events, including whether and how a demonstration will take place, how many people participate, and how those in power (e.g., state, military) respond. Similarly, these media platforms alert us to more global shifts within a conflict, such as when fighting moves to a new city or region, the number of casualties and wounded, and the types of weaponry used by parties [5]. In monitoring social media channels, government forces may respond to the perception that citizens are gaining strength and sympathy. Governments have been shown to be quite tolerant of dissent so long as it does not metamorphose into collective action [20].

Protestors and social activists communicate with each other with increased transparency due to the ubiquitous availability of social media like Twitter. Activists are able to access these tools via mobile phones and computers with Internet connectivity, providing a stream of real-time shared information. Citizens can share the location of events like protests and military incursions and the degree of state repression. In turn, the government—interested in remaining in power—monitors social media platforms to ascertain emerging threats [12], such as where new groups are forming, where existing groups are strengthening, where gatherings are held, and who the leaders are.

With few exceptions, leader Bashar al-Assad chose to keep social media platforms up and running. Gohdes [21] notes that regimes can attempt to thwart the growth of social protests online and in the streets by shutting down the Internet. During these times, military repression increases, but government information about the citizens decreases. As a result, regimes may elect to maintain Internet connectivity to monitor and surveil their citizens.

At the same time, the insulation of online social platforms allows researchers to detail real-world social dynamics at unprecedented resolution and scale [22]. The digital traces left by individuals and collectively as groups are regarded as a valuable source of information that can offer powerful insights into states of social disequilibrium, uprisings, and escalations into civil wars. By examining these conversations, researchers can study the degree of cohesion between speakers and the alignment between that cohesion and their collective political behavior, just as the degree of cohesion among real-world protestors in past movements has been captured in their shared sign messaging [3].

### The role of cohesion in social movements and civil wars

People join social and political resistance movements because they feel a sense of connection to the cause. Given that people are generally risk-averse, overcoming inertia requires a strong connection to the group identity or a strong aversion to an individual, event, or political injustice [23, 24]. Political resistance offers participants the opportunity to “lose themselves” and relinquish their individual identity in favor of identification with a larger group, contributing to a cohesive group ready and mobilized for action [25].

The type of collective contentious behavior that manifests in society is a function of the available political opportunity structures. The political opportunity structure is “comprised of *(sic)* specific configurations of resources, institutional arrangements and historical precedents for social mobilization, which facilitate the development of protest movements in some instances and constrain them in others” [26, p. 58]. Emergent strategies and tactics in contentious politics are a function of what the system makes possible. This includes the number of people and their disposition (i.e. nonviolent or violent), as well as their internal structure. The more cohesive a group is, the greater threat they pose to the establishment.

Cohesion is a familiar concept in the study of civil conflicts, as it pertains to group affiliation and the ability to overcome collective action problems. Individuals must act in concert—that is, cohesively—to accomplish the task of challenging state authority [27–30]. Viewing rebel recruitment during civil conflicts as a function of internal cohesion, rebel movement success is influenced by the degree of group cohesion, which itself is a function of geographic distance, ideology, and ethnicity [31]. Within Syria, multiple rebel groups exist, sourcing fighters locally (from the disaffected Syrian population) and internationally (from transnational terrorist organizations). Groups compete intensely with one another for recruitment in the rebel movement and often punish defectors for nonparticipation [31].

Another factor influencing social cohesion during social movements and civil wars is ethnic identity and the degree of ethnic conflict associated with the movement. Greater degrees of ethnic conflict increase the salience of ethnic identity, leading people to identify more strongly with their own ethnic identity [32]. As ethnic conflict intensifies, groups seek to become more quintessentially like the typical member of their ethnic group. This can manifest in a number of ways, including in behavior on social media. However, this may also include a general identification across ethnic groups in a membership of an opposition to an oppressive leader.

The civil war literature has also explored the role of social cohesion. Social homogeneity increases the likelihood of social conflict, especially when the society is polarized [33]; this is partly due to nationalist groups’ strong identification with a collective identity [34] and their social solidarity [32]. These same processes can be similarly important for rebels [35]. Referencing Gurr’s [36] work on minorities at risk, Regan and Norton [37] note that a religious or linguistic minority might suffer disproportionately in a given society, and this form of aggrievement can lead to unrest across the social lines that distinguish the minority group. Therefore, while collective cohesion is a function of targeted maldistributions, it is individual penury that allows for this collective mobilization. (p. 323)

The body of previous work concurs that group cohesion matters to group mobilization. We build on this by providing a quantitative approach to understanding cohesion from multiple levels and by incorporating analytic tools that allow us to investigate the interconnectedness of those levels.

### Cohesion: A multi-leveled, multi-layered concept

We contextualize linguistic cohesion with social mobilization and response to the relative intensity of the conflict. Cohesion is a multi-layered concept referring to the connectedness of speakers, actions, and societies. It reflects the continuity of language and expression and shows the connectivity of participants in a social movement. To address the relationship between language and discourse and social movements, we adopt an interdisciplinary approach that incorporates linguistics, psychology, and political science using nonlinear dynamical systems methodology to disentangle the relationship between language and action.

#### Lexical and interlocutor cohesion

Just as real-world social cohesion can be quantified, so can online cohesion. One kind of cohesion is the similarity in language use on social media. This implicit or explicit similarity can indicate shared beliefs, thoughts, and feelings, as has been studied in research on language style in psycholinguistics and social psychology [38–40]. Although previous work on language style has often focused on individuals and small groups, this can be scaled up to examine massive social-level changes [41, 42].

**Fig 1:**
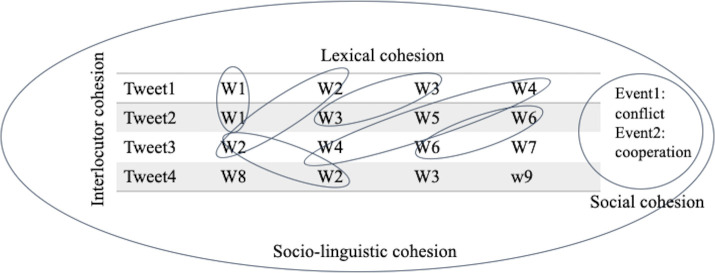
A visual representation of cohesion in tweets. The shared content words–either identical or semantically similar–constitute a theme around which cohesion is occurring.

The first level of cohesion that we will consider are the words within social media posts. In Fig 1, we see four sequential tweets of four words each. Each sequential tweet contains some content included in the previous tweets. The linguistic construct of content word overlap measures the relationship across units of meaning—here, tweets—where identical and semantically similar words are repeated [43]. These repeated lexical items constitute a theme; on Twitter, this might be a particular hashtag, a name, a location, or another nominal category. This localized cohesion is referential; words are repeated and co-referred across tweets, creating a sense of continuity and cohesiveness.

Typically, these analyses focus on *content words* (e.g., nouns, adjectives, verbs), which convey more meaningful semantic information than *function words* (e.g., articles, conjunctions). While function words can provide important information about complexity and language structure [40], content words have more specialized meaning and are therefore more useful when researchers want to understand *what* people are discussing, rather than *how* they are discussing it [43, 44].

In estimating cohesion in Twitter posts, tweets that share content words yield higher cohesion values, even among speakers with opposing views on the subject matter. Repetition of terms and concepts is captured by reiteration, which is a type of lexical cohesion in which a subsequent item repeats a previous item, or refers to it through synonymous language [45]. We include retweets (i.e., reposts or forwards of previous messages) because they are a form of reiteration that increases message cohesion. They indicate that individuals are “singing to the same tune” by amplifying the original message. Since retweets replicate verbatim another tweet (sometimes with the addition of commentary or supplementary messages), these tweets would also have higher cohesion.

We also include hashtags in our analyses. Hashtags are a way of identifying similar topics (e.g., people, events, places, themes) across users and across tweets by typing a string of characters preceded by a “#” symbol. Users may choose to include as many hashtags (up to the total character limit of the tweet) as they would like, and they may choose to create their own or adopt hashtags from other users or groups. The use of the same hashtags would increase cohesion; however, because hashtags are often included or embedded within longer messages, simply using the same hashtags would not be a guarantee of higher cohesion if the rest of the words in the tweets do not overlap.

The use of similar language can provide insight into *interlocutor cohesion*, or the degree to which people are talking about similar things. This can include people who are talking about similar things from radically perspectives, as well as those who are talking about something from the same perspective. Previous research from political science has investigated these ideas [3]: The “Occupy Wall Street” movement has been held up as an example of a movement with low interlocutor and linguistic cohesion because of its wide range of signs and slogans, while the “I am a man” protest (during the 1968 sanitation workers’ strike in Memphis, TN) or the “Black Lives Matter” placards (following the uprising in Ferguson, MO) have high lexical and interlocutor cohesion [46].

Fed by lexical cohesion, interlocutor cohesion ebbs and flows, contextualized by changing real world events. Interlocutor cohesion may increase preceding an in-person social mobilization event, alerting a repressive government that collective mobilization is happening. It may also respond to exogenous events such as spontaneous, unprovoked militarized conflict.

Given the importance of lexical and interlocutor cohesion for messaging in political movements, lexical cohesion may be important trace data for researchers interested in leveraging social media data to analyze social movements. While overall Twitter activity (i.e., pure volume of tweets or retweets occurring each day) can provide insight into the level of engagement and its relationship to real-world events, the quantification of linguistic cohesion across tweets allows us to investigate if the platform is being used *toward a particular end*. A day with a high volume of tweets may or may not be focused on a particular topic, but a day in which many tweets include the same phrase points to a shared event, movement, or social experience. This coalescence of many tweets around a topic—regardless of the *side* of the topic of any given tweet—indicates a matter of high social relevance. By quantifying the collective focus of users, we gain insight into their intentions, rather than their general level of engagement.

Similar to large-scale studies of sociocultural movements (sometimes referred to as *culturomics*; e.g., [42]), political scientists can track the ways in which conversations on social media coalesce or diffuse around similar content words. In this way, it matters relatively little whether the interlocutors have precisely the same views: The point is to understand whether the society-level conversation is converging on similar themes.

#### Social and political cohesion

Real-world collective action is known to bring about a sense of togetherness, also known as “collective effervescence” [47]. This feeling of being a part of something larger than oneself leads individuals to act in ways that would not have on their own. Being overcome with this feeling can lead to violence [48, 49]. Conversely, increased exposure to violence can result in positive social cohesion. Gilligan, Pasquale, and Samii [50] found that exposure to fatal wartime violence resulted in increased prosocial motivations, including cooperative behaviors, feelings of trust, and overall community-level social cohesion.

At the same time, behavioral similarity in certain contexts has been connected to important psychological and social effects. For example, shared language is associated with rapport (both in face-to-face and computer-mediated conversations; [51, 52]) and can even be connected to better performance on shared tasks (as long as the language is relevant to the task at hand; [53]). Shared movement is associated with greater group bonding and greater willingness to contribute to the group [54], and shared identity leads to a greater willingness to adopt the group’s goals and motivations [55].

We expect to see increased linguistic cohesion among people participating in online discussions about an idea, event, individual, or movement. While the overall frequency of social media messages related to the event may increase as a result of the event, their linguistic cohesion helps us understand if denizens are using the platforms in a coordinated way that amplifies their messages or goals and creates a virtual protest space. Given that Syrian protesters coordinated their activity through Twitter by discussing their own events (e.g., planned protests, active demonstrations) and their reactions to events happening around them (e.g., government reactions, international action or inaction), the cohesion in social media should correlate with the social cohesion of the group [56].

Political and social cohesion in the world can be modeled using event data which encodes who does what to whom on a given day. The CAMEO (Conflict and Mediation Event Observations) coding scheme encodes events and activities with numerical values, rescaled to reflect a conflict-cooperation scale [57, 58]. These event codes include a range of behaviors of varying intensities and valences, from making public statements and appeals to demanding, threatening, protesting, and using unconventional mass violence.

Social cohesion can have real and wide-ranging impacts on a political movement and the people in it. A socially cohesive group is inherently more threatening to the existing regime because it reflects momentum in collective opposition and the professionalization of alternate leadership. Global insurgency movements exhibit similar patterns in their social structure [59]. Rather than patchwork messages via Twitter, emergent or intentional cohesive online messaging—using similar language, paralleling in-person messaging [3]—demonstrates increased organizational structure, inciting a government response.

Thus, lexical and interpersonal cohesion are hypothesized to be tied to social and political cohesion. Shared online language may lead to the development or expression of shared identity [54]. This, in turn, may drive individuals’ propensity to engage in coordinated behavior for the group [54, 55]. Taken together, these processes may increase movement participants’ weak and strong ties [1, 18, 19], building the movement by expanding and deepening heterogenous ties. Therefore, during social conflict, the linguistic cohesion of the online conversation should correspond to the degree of social cohesion on the ground.

Steinert-Threlkeld [4] modeled a similar relationship between social media and social mobilization alongside event data. That groundbreaking work demonstrated that participants on the fringe of a social movement contribute substantially to the overall level of protest. Here, in addition to providing new methods to this area, we build on this work by demonstrating how sociolinguistic cohesion evolves in tandem with negative and positive exogenous events.

#### Sociolinguistic cohesion: Connectedness of speakers, ideas, and events

**Fig 2:**
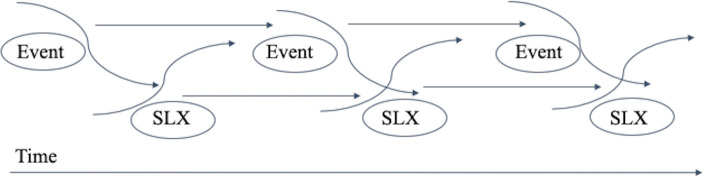
The theoretical framework for sociolinguistic cohesion. Real-world events and linguistic cohesion continually interact and influence one another throughout time.

We tie the constructs of lexical, interlocutor, and social cohesion together in a theoretical framework called *sociolinguistic cohesion*, as shown in Fig 2. Previous values of linguistic cohesion should influence future values of linguistic cohesion and future actions in the real world space. Previous events influence future events and future linguistic cohesion on social media.

Sociolinguistic cohesion is not a static concept. Just as social movements experience increased participation and setbacks—indicating dynamic relationships among participants and between participants and the sociopolitical environment—sociolinguistic cohesion is also dynamic, reflecting the changing states of the political sphere. Other scholars have noted the diffusion of messages that reach critical mass, allowing for “behaviors, beliefs, and preferences” to spread within a population [60]. Cohesion changes over time in response to external events and the internal dynamics of the social movement, even if the social interaction occurs entirely online [61].

Lexical cohesion, then, is a useful indicator of social cohesion in political crisis. Lexical cohesion happens at the word level. Interlocutor cohesion happens between speakers. Political cohesion happens at the social level during moments of political crisis, like civil wars and social movements. Sociolinguistic cohesion comprises lexical, interlocutor, and sociopolitical events, in which the words and their context reveal the disposition of socially connected linguistic communities. It accounts for the cohesion between multiple speakers in an environment as their language becomes more similar. Sociolinguistic cohesion also addresses how groups involved in political rebellion converge to mount sustained resistance to state authority.

Related previous work has also explored diffusion of hashtags in viral contagion in Nigeria, a phenomenon similar to what we identify as sociolinguistic cohesion [62]. The prevalence and rate of use of particular hashtags contributed to the overall sociolinguistic cohesion. The more that people reuse hashtags or common phrases, the greater the sociolinguistic cohesion when this phenomenon occurs during socially significant times like protests [63].

Combining these separate threads, it is reasonable to expect that real-world actions and online social interactions would be enmeshed, driving and being driven by one another. From a dynamical systems perspective, online social cohesion and in-person action can be considered two complementary components of a single system–constantly constrained by, interacting with, and influencing one another [64]. Investigation of the coupling of real-world mobilization and social cohesion could provide new insight into the power of social media during mobilization and the ways in which individuals utilize it to bring about, or respond to, social change. Increasingly, social media platforms are being utilized during social mobilization [65, 66], but the relationship between virtual and real-world mobilization is still largely unknown. The present work aims to understand how physical and virtual movements coexist and interact from a dynamical systems perspective.

### Using nonlinear methods to capture complex relationships

Given the hypothesized interrelationship between the real-world and online actions—and the notorious messiness of naturally occurring data—it is critical to find appropriate analysis tools that can identify the complex interconnections. Traditional linear analyses have led to many notable discoveries in the domains of psychology and political science, but time series data pose problems that are not easily overcome with these methods. Events have historically been conceptualized as linear chains of smaller, sequential events with one occurrence leading to the next. Certain kinds of data are relatively well-suited to these analyses, but messy, complex, variable data from real-world events can pose challenges, from overpowered samples to violation of underlying statistical assumptions [22].

At the same time, the foundational theoretical assumptions of a linear world have been increasingly replaced by ideas from dynamical systems theory. Rather than a chain of events, time series data can be thought of as nested occurrences, where mutually constrained parts (e.g., citizens, governments, real-world events, social media platforms) are constantly interacting, leading to more global behaviors that are larger than the sum of the parts [67]. When analyzed as dynamical systems, many phenomena exhibit new nonlinear patterns that were formerly overlooked. Nonlinear methods provide means for capturing the rich temporal dynamics and variability that unfold in time series data.

Although a number of other nonlinear methods exist, the primary nonlinear methods used in the current work are cross-recurrence quantification analysis (CRQA; [68]) and its offshoot, windowed cross-recurrence quantification analysis (WCRQA; [69]). CRQA has successfully been used to gain a better understanding of many complex human phenomena, including a variety of human social interactions (for a review, see [70]). Much as it has for psychology, we argue that the use of nonlinear methods in the investigation of political science phenomena will lead to new insights and the development of more robust theories.

### The present work

Social media has become a primary platform for social movements and activism across the world, but the extent to which virtual activism reflects and drives real-world mobilization is still largely unknown. In the current work, we conceptualize the physical and virtual movements as a dynamical system, both constantly interacting with and influencing the other. We aim to study this by analyzing the real-world events and online social cohesion during nearly three months of the Arab Spring. Both real-world and virtual collective coordination were powerful tools in the events of the Arab Spring and are undeniably linked. This study aims to explore the dynamics of Twitter users in Syria, who used the social media platform to raise awareness about the Arab Spring and the relationship of those messages to mass mobilization in the real world.

Using cross-recurrence quantification analysis (CRQA; [68]) and windowed cross-recurrence quantification analysis (WCRQA; [69]), we analyzed the co-evolution of social cohesion online and real-world events. Specifically, using heterogeneous data from March 31, 2012, to June 15, 2012, we compared the social cohesion of Syrian tweets with the counts of all, positive, and negative real-world events derived from international event data (i.e., the Integrated Crisis Early Warning System; [71]).

We hypothesized that the count of all events and the count of negative events would show high levels of coupling, as this time period was marked by daily violence which often leads to increased salience. We also hypothesized that the count of positive events would not show patterns of coupling, as these events were likely overshadowed by the salient negative daily occurrences. After testing these hypotheses, we then conducted exploratory analyses to further examine the patterns identified with CRQA. Using WCRQA, we sought to identify shifts in the dynamics of the systems that could then be linked to real-world shifts in conflict frequency. Through these analyses, our two primary goals in the current work were to uncover (1) how different intensities during times of turmoil may enhance or deter social cohesion in social media communication and (2) how social mobilization may be evident in the dynamic coupling of cohesion and real-world events.

An additional focus of the current work is to introduce cross-recurrence quantification analysis—a longstanding method from physics that has since become influential in a variety of other fields—to political science. We do so by providing detailed descriptions of the methods and providing clear directions about interpretation and significance testing even with relatively small-*n* or case studies. After demonstrating these methods’ utility with this dataset, we close the paper with specific suggestions to political scientists interested in these methods.

This work is a reproducible manuscript. Code and analyses can be found at: www.github.com/mchiovaro/arab-spring.

## Method

### Materials

#### Tweet corpus

The corpus of tweets used included 3443742 English-language Syrian tweets occurring between March 31–June 15, 2012. Using the Twitter streaming API, servers at the University of Texas at Austin collected the tweets. The servers were run on a stable (LTS) version of Ubuntu Linux (v. 10.04) and were connected directly to the high-bandwidth UT network maintained by IT services.

Tweets collected contained one or more of the following keywords: “syria,” “syrian,” “damascus,” “homs,” “al-assad,” and “sunni.” These keywords were chosen based on the most distinctive terms used in news articles covering the Arab Spring in Syria at the time. A node.js script was used to reinitialize the connection to Twitter’s servers whenever the script timed out due to a period of silence. The script requested data from the API endpoint using basic HTTP authentication (OAuth was optional, at the time) at https://stream.twitter.com/1.1/statuses/filter.json and stored the data directly to the local hard disk. This collection method complied with the terms and conditions of the Twitter API. The tweets were then post-processed with a Python script to convert the JSON-formatted tweets to tab-separated files, which were then compressed to maximize storage efficiency.

The corpus consisted of 63.2% original tweets, 33.5% retweets, and 3.4% replies. There were 574104 users in the corpus and an average of 44,723.92 tweets per day.

Scripts used for collecting tweets and compressing data can be found at https://github.com/chbrown/twilight.

#### Event data

Event data were obtained from the 2012 Integrated Crisis Early Warning System (ICEWS; [71]). The event data were first filtered to remove incomplete and incorrectly formatted data. They were then filtered to include only events that were directed at (i.e., target) Syria (n = 6300). To explore the impact of outwardly directed Syrian events, the data were filtered again to include events that were both directed at (i.e., target) or directed by (i.e., source) Syria (n = 7990).

### Data preparation

#### Social cohesion metric

The social cohesion or linguistic similarity of daily tweets was calculated by quantifying the frequency of shared content words with respect to those words’ frequencies in the entire tweet corpus. Retweets were included in the corpus and used in the social cohesion calculation, as they indicate strong support for an individual’s message. Stop words and duplicate words were not removed from the tweets, while punctuation marks were. The lengths of tweets were disregarded, as the aspect of interest is in shared linguistic content and not breadth of description. The presence of bots was not investigated. However, according to a 10-Q report for Twitter, Inc., for a quarterly period in 2014, the estimated number of third-party applications automatically contacting the servers (i.e., bots), was less than 8.5% [72]. As the data in this study were collected two years prior, this number was likely even less. Thus we consider the number of bots negligible for the present investigation.

The tweets were first sorted into ascending order by timestamp. They were then grouped into successive sets of five tweets. Groups of five were chosen to allow for manageability and efficiency of the analyses. Linguistic cohesion was quantified for all possible pairs of tweets in the group of five, resulting in 10 pairs per group of tweets; all 10 cohesion values for the group were averaged for a single group cohesion value. The cohesion value for each day was computed as the average cohesion values for all groups whose first tweet was on that day. Each pair’s cohesion value was calculated in three parts:

$$Inverse\;frequency\;weighting\;of\;overlap\;words=\sum_{t_{i}\in d_{1}\cap d_{2}}\frac{\log\left(f(t_{i},d_{1})+f(t_{i},d_{2})+1\right)}{\log\left(f(t_{i})+1\right)}$$ (1)

Eq 1 identifies the inverse frequency weighting of each overlapping word *t*_*i*_ between two tweets *d*_1_ and *d*_2_, relative to the word’s frequency in the corpus D, for all overlapping words *t*_1…*k*_ between the two tweets.


$$Inverse\;frequency\;weighting\;of\;all\;words=\sum_{t_{i}\in d_{1}\cup d_{2}}\frac{\log\left(f(t_{i},d_{1})+f(t_{i},d_{2})+1\right)}{\log\left(f(t_{i})+1\right)}$$ (2)

Eq 2 then calculates the inverse frequency of each word *t*_*i*_ in tweets *d*_1_ and *d*_2_, relative to the word’s frequency in the corpus D, for all words *t*_1…*n*_ that appear in either tweet.


$$Two\;{tweets}^{\prime }similarity=\frac{inverse\;frequency\;weighting\;of\;overlap\;words}{inverse\;frequency\;weighting\;of\;all\;words}$$ (3)

Finally, Eq 3 identifies their linguistic cohesion by taking the ratio of the results of Eqs 1 and 2. Through this process, we are able to naturally account for the frequencies of natural language generally and of online language specifically: Words that occur many times in the corpus (which we can operationalize as function words; e.g., prepositions, pronouns) influence the similarity scores between two tweets much less than words that occur rarely in the corpus (which we can operationalize as content words; e.g., names, hashtags).

**Fig 3:**
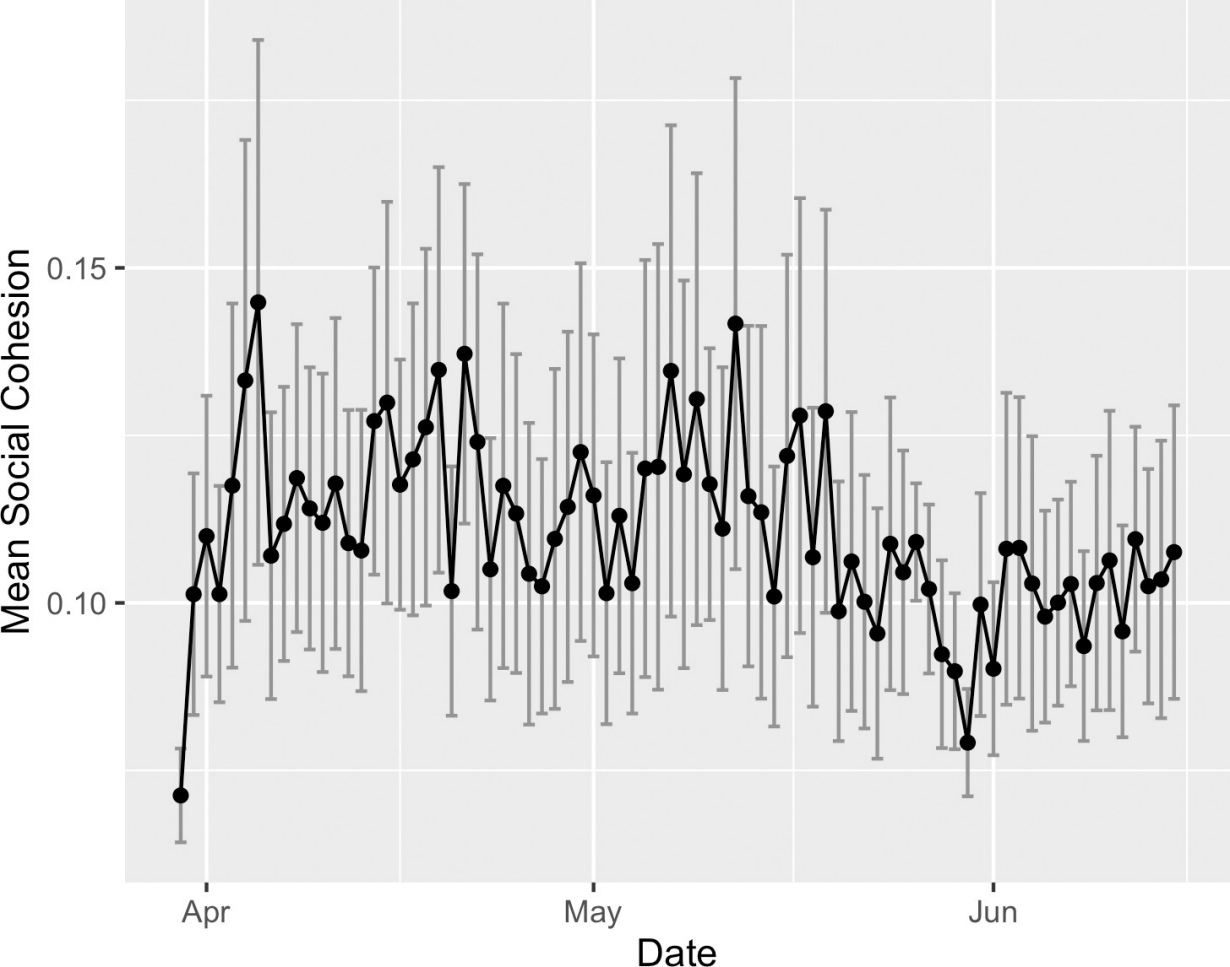
Raw social cohesion metric time series.

This linguistic cohesion metric is similar to *term frequency-inverse document frequency* (tf-idf; see also [73, 74]). However, the method focuses more squarely on terms (i.e., agnostic to the number of documents in the corpus), aggregates at a higher semantic level (i.e., summing across words), and accounts for linguistic novelty relative to the pair and the corpus (i.e., ratio of Eq 1 to Eq 2). The social cohesion calculation was performed on the tweet corpus containing English tweets. All hashtags were included in the analysis, while URLs were removed. The raw social cohesion time series can be seen in Fig 3.

### ICEWS event time series

Coming from the Integrated Crisis Early Warning System (ICEWS) developed at the Defense Advanced Research Project Agency, the daily events used were scored with an intensity rating ranging from -10 to 10. Negative events—such as riots and attacks—are coded with low negative numbers, while positive or peaceful events are coded with high positive numbers. This well-validated coding scheme allows for numerical quantification of different event types.

For both the target-only and the target and source data described above, time series of the count of all events were generated by summing the total number of events that happened each day, irrespective of ICEWS score. Similarly, time series of count of positive events were generated by summing the number of ICEWS events for each day that had an intensity rating greater than zero. Lastly, time series of count of negative events were generated by summing the number of ICEWS events for each day that had an intensity rating less than zero.

**Fig 4:**
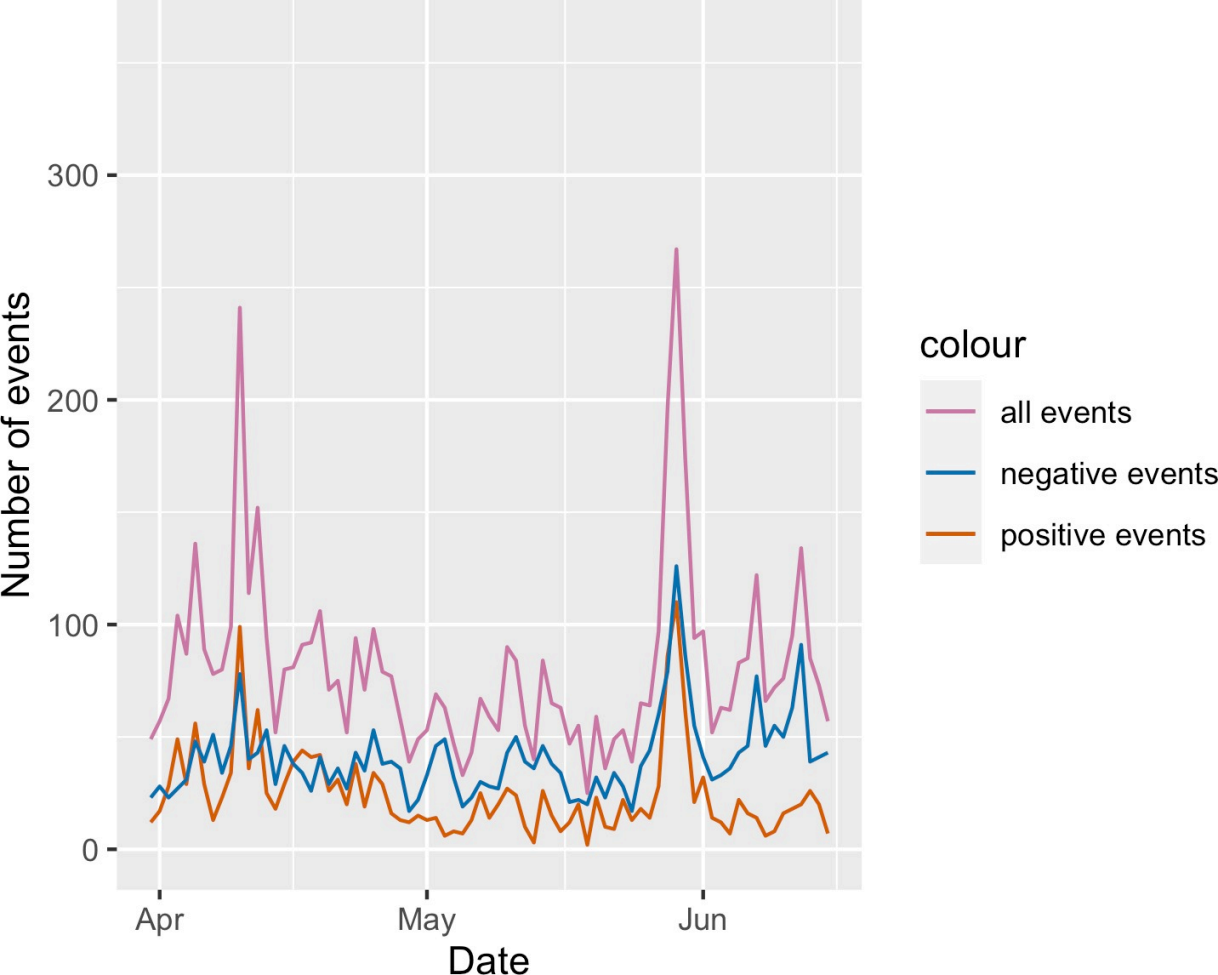
Raw event count time series for target filtered data.

**Fig 5:**
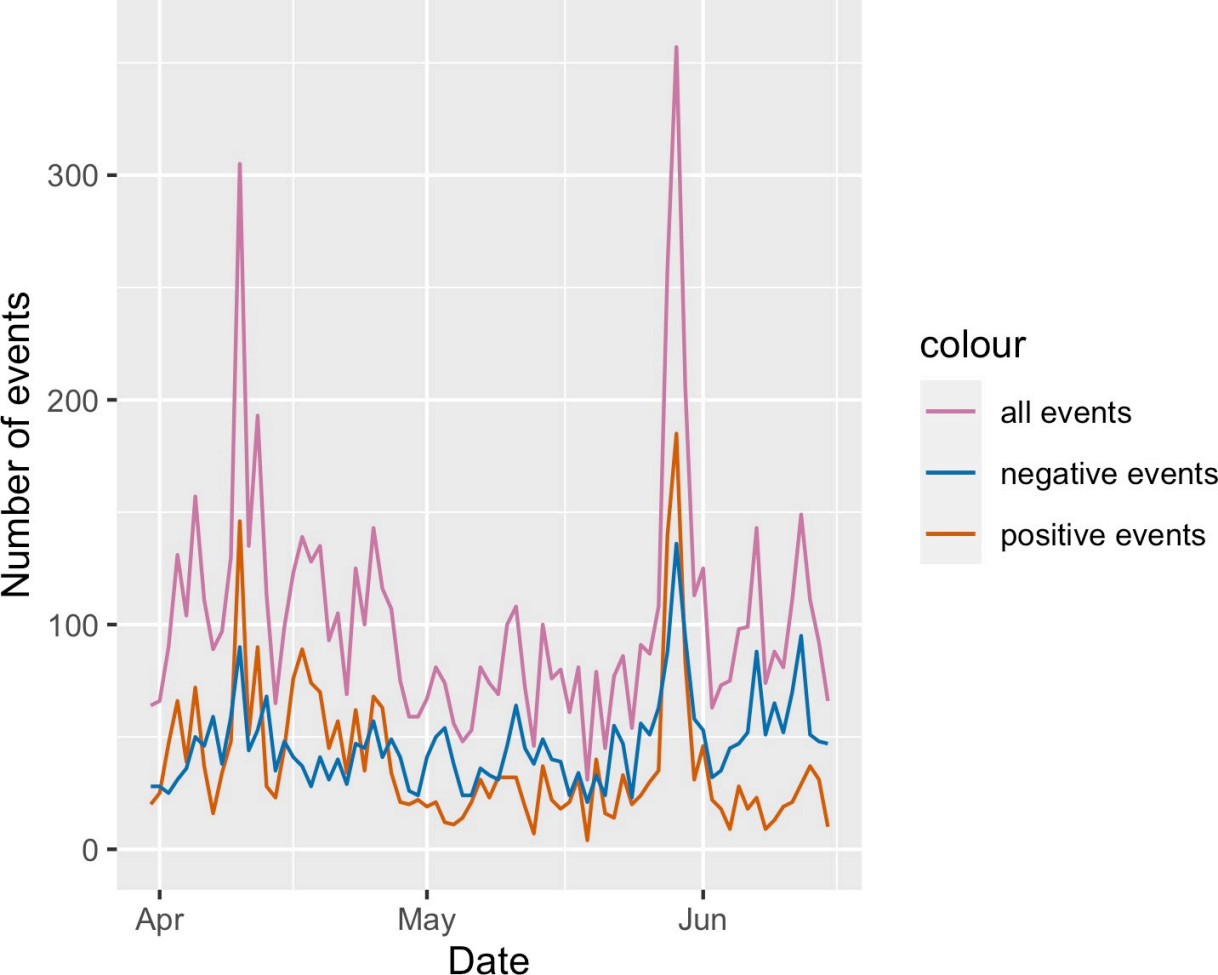
Raw event count time series for source and target filtered data.

The raw event time series for target filtered data can be seen in Fig 4. The raw event time series for source and target filtered data can be seen in Fig 5.

### Analyses

#### Cross-recurrence quantification analysis

We first used categorical cross-recurrence quantification analysis (CRQA; [68]) to investigate the temporal patterns of social cohesion and real-world events in Syria during the Arab Spring. CRQA is an extension of RQA, which captures the patterns of revisited states of a single time series [75, 76]. CRQA allows for two time series to be projected onto one another to investigate shared states and trajectories. In CRQA, we track these shared states by identifying *recurrent points*, or intersections of time during which two signals shared the same state. These shared states can be at the same time (i.e., in synchrony) or at later points in the time series. We can further chart shared trajectories by examining the occurrences and patterns of multiple sequential recurrent points.

**Fig 6:**
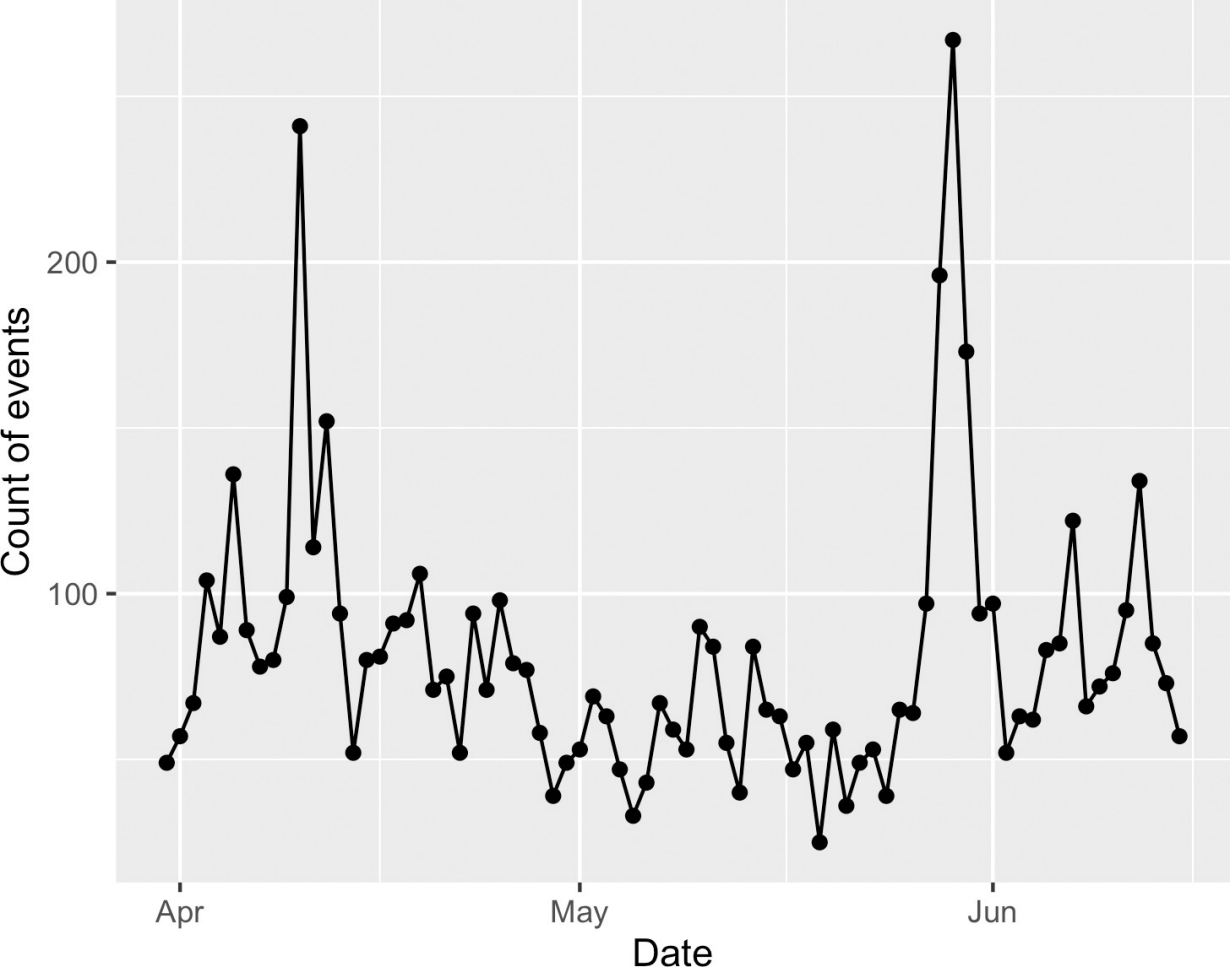
Example of pre-processed data.

**Fig 7:**
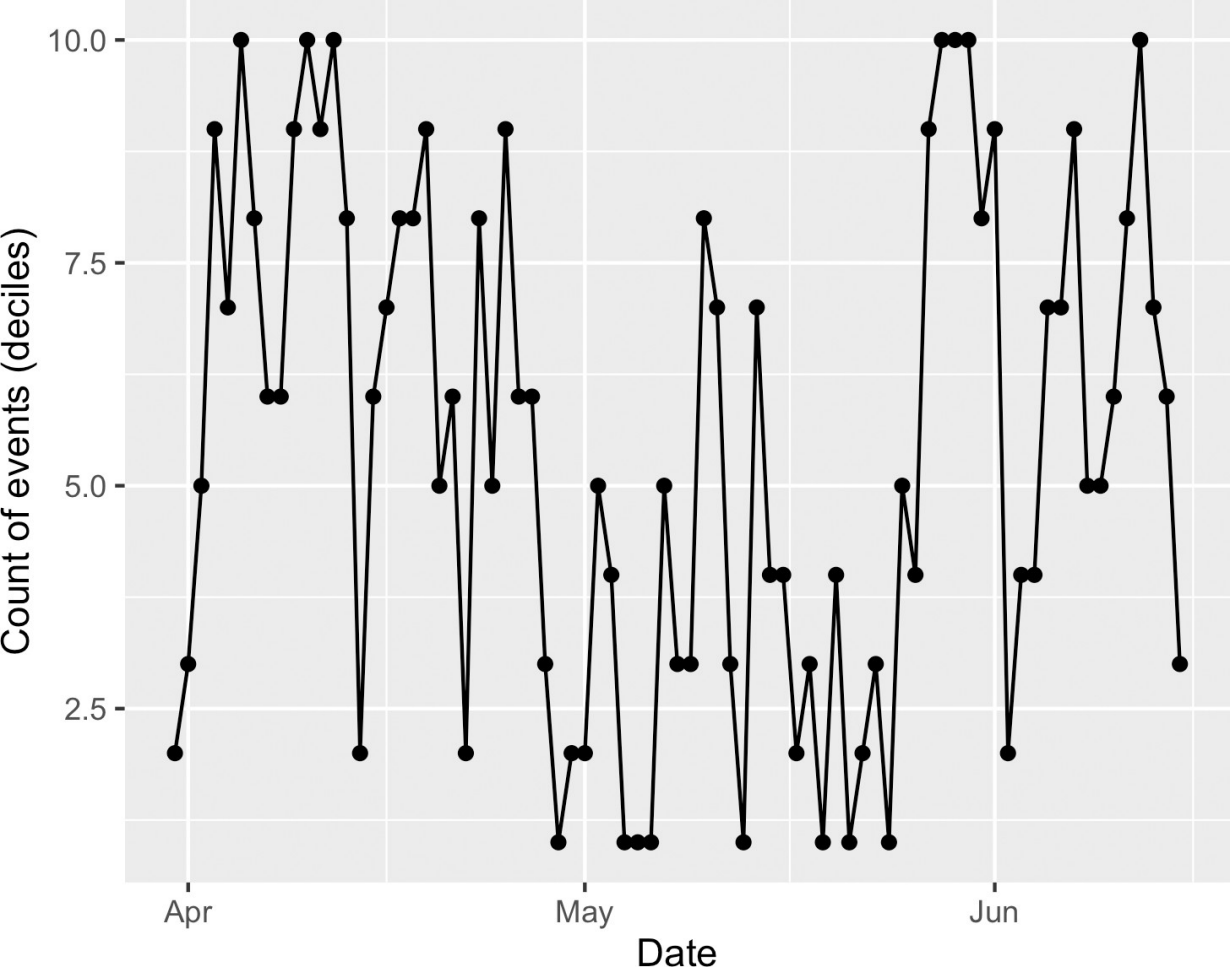
Example of deciled data.

To perform categorical CRQA, the time series must be converted to the same scale, while still preserving a sampling rate that respects the native timescale of the systems (cf. Nyquist frequency in signal processing; [77]). In other words, a system that changes every day must be sampled more often than a system that changes every year and less often than a system that changes every hour. However, to compare two time series with CRQA, the two time series must be sampled at the same frequency as one another. Because the social cohesion and event data have different scales and values, we converted each time series of daily values (e.g., Fig 6) into deciles (e.g., Fig 7) to analyze the relative dynamics of different levels of social cohesion and counts of all, positive, and negative events on each day. We implemented CRQA using the crqa package [78] for R [79].

*Visualization*. A cross-recurrence plot (CRP) is the visual representation of CRQA. To construct a cross-recurrence plot, two time series are plotted against each other on the *x* and *y* axes. At every point where they share the same state, a point is placed on the plot to indicate recurrence. (For categorical CRQA, this process is similar to the Cartesian product.)

**Fig 8:**
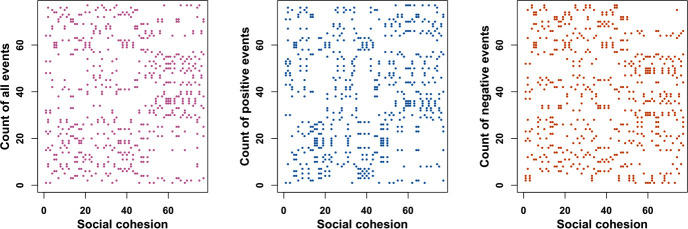
Recurrence plot (RP) for social cohesion and counts of events from target filtered data.

**Fig 9:**
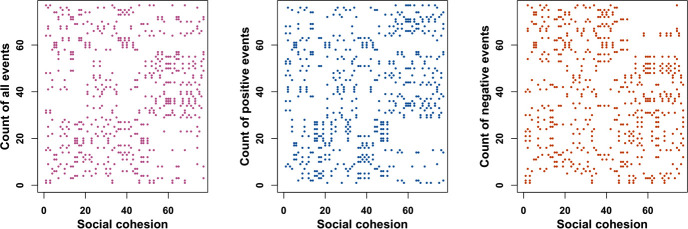
Recurrence plot (RP) for social cohesion and counts of events from source and target filtered data.

We can visually analyze these plots to reveal patterns of structure and recurrence. The main diagonal line on the plot is known as the *line of synchrony*. This line falls where the time series match temporally. A completely filled main diagonal line would indicate perfect in-phase synchrony between the two time series. See Figs 8 and 9 for examples of RPs.

*Metrics*. While visual inspections can be informative, we can derive a variety of quantitative metrics from the cross-recurrence plot. Each of these metrics provides information into the patterns and dynamics of the new shared system. These metrics are also critical for inferential statistics. We will focus most directly on two of these metrics: *recurrence rate* and *determinism*.

The percentage of recurrent points to total possible points is a metric called the *recurrence rate* (*RR*). This metric gives a general description of how frequently the time series are sharing the same states. In the current study, a recurrent point would indicate that the level of social cohesion and frequency of events were at the same intensity for two particular points in time (in this case, one-day windows). Importantly, the nature of CRQA allows us to quantify how a system shares similar states across time: In other words, it identifies recurrence whether a window of shared intensity occurs on the same day for the two time series or after a one-week gap between when the two time series exhibit the same intensity.

Recurrent points that occur in successive moments form line structures. These are conceptualized as shared trajectories, where the two time series are moving together across time in a shared state. The percentage of recurrent points on the plot that fall along diagonal line structures (i.e., two or more consecutive points) is a metric called *percent determinism* (or simply *determinism*; *DET*). For the current project, a high DET would indicate that not only are the levels of social cohesion and frequency of events occurring at similar intensities, but they are doing so across consecutive days.

A number of other metrics provide insight not only into the amount of structure between the systems but about the nature of this shared structure. The average length of the line structures is called *mean line* (L), which can be conceptualized as the average amount of time that the two time series move together before one or both shift states. The longest line length is called *max line* (*maxL*); it indicates the longest instance of being “stuck” in a particular state together and is a measure of the stability of the system. The total *number of lines* on the plot (*NRLINE*) scales with structure: The more lines that exist, the more coupled the two systems are. The complexity or variability of line lengths is captured by a metric called *entropy* (*ENTR*)—which may also be normalized by the total number of lines (i.e., *normalized entropy*; *rENTR*)—and indicates the stability and predictability of the system. When one of the signals gets “stuck” in a state and the other moves on to other states, this results in vertical line structures, which are quantified in *laminarity* (*LAM*; i.e., the proportion of points forming vertical line structures) and *trapping time* (*TT*; i.e., the average length of the vertical trajectories). For more detail on CRQA metrics, see [78].

*Parameters*. To conduct the analyses on the decile data, the parameters were set to those typically used in categorical CRQA: Delay was set to 0, embedding dimension was set to 1, and radius was set to .001 to allow for only exact recurrent matches. No normalization was done, and lines were considered two or more consecutive recurrent points. The Theiler window was set to 0 to include points along the line of synchrony. For more details on the CRQA parameters and how they impact analyses, see [78].

*Approximate permutation test*. Unlike some nonlinear analyses (e.g., fractal analyses), CRQA metrics do not have inherently meaningful values. As a result, CRQA is best used as a relative metric—that is, comparing the CRQA values across conditions or subsets of the data. Typically, meaningful differences between values are determined with traditional inferential statistics. While this may not be a problem for experimentally derived datasets, it can pose a barrier to researchers using smaller datasets or conducting data-informed case studies.

For CRQA, this can be addressed using *approximate permutation tests*. Similar to more established methods of statistical significance in CRQA research (which create surrogate time series; e.g., [80]), the permutation test approach to significance testing allows the researcher to use a time series as its own baseline by testing whether the *structure* of the unfolding events of the time series—rather than the *raw frequency* of the events within the time series—cohere together more than would be expected by chance. Permutation tests are used to identify null distributions for test statistics when the null hypothesis is that the group assignment does not matter, and they can be used when traditional parametric tests may not be appropriate (e.g., [81, 82]). The test is similar to bootstrapping, except that the sampling of observations is done without replacement.

To quantify the differences in the CRQA metrics from those that would be expected by chance, we conducted approximate permutation tests for significance with upper and lower bounds for the 95th and 99th percentiles (comparable to traditional alpha criteria of .05 and .01, respectively). In this approach, we simply create a large number of permutations of the original time series (i.e., breaking up the dependency across time points but preserving the frequencies of the events), conduct CRQA for each of those permutations, and then examine whether the real time series’ CRQA metrics exceed those that we would expect to see by chance. The proportion of times that the real time series’ values exceeds the surrogate time series’ values can be interpreted as the alpha criterion to determine significance. For more information on permutation tests, see Good [83].

Using the real deciled time series of social cohesion, count of all events, count of positive events, and count of negative events, we conducted a series of permutation tests (one for the comparison between social cohesion and each type of event count) to determine whether the observed measure is statistically different from chance for each CRQA metric. Using the sample function in base R [79], 1000 permuted time series were created for each of the real time series. CRQA was then run for each permuted time series combination for social cohesion and count of all events, social cohesion and count of positive events, and social cohesion and count of negative events using the permuted time series. This resulted in 1000 results for each of the CRQA metrics for each combination of time series type. Statistical significance from chance was set at the 95th and 99th percentiles of the metrics from the permuted CRQ analyses. Given that a permutation test is similar to bootstrapping but does not include replacement, all real recurrent points were included in each CRQA analysis, leaving RR constant across all permutations. Thus, RR is not included in the inferential tests.

*Windowed cross-recurrence quantification analysis*. We used windowed cross-recurrence quantification analysis (WCRQA; [69]) to further investigate the evolution of the temporal dynamics of social cohesion and real-world events. In this analysis, a smaller “window” of time points is used to calculate CRQA and capture more fine-grained dynamics of two time series. The window remains the same size and “slides” diagonally up the line of synchrony. The changes in the CRQA metrics across the windows can reveal changes in dynamics that can then be linked to external, real-world events. We implemented WCRQA using the crqa package [78] for R [79].

*Metrics*. For WCRQA analyses, we decided to focus on RR and DET, but similar analyses and visualizations can be performed for any RQA metric.

*Parameters*. In addition to the CRQA parameters, two other parameters are required when conducting WCRQA. First, the *window size* will determine how many observations are used in each CRQA analysis. We conducted WCRQA with a window size of 14 to investigate the dynamics over two weeks and allow for the capture of more structure and thus a more meaningful interpretation of DET. We first attempted to use a window size of 7 to investigate the dynamics at the week scale, but found that window sizes smaller than 14 limited the possibilities for shared trajectories and made determinism uninterpretable. In addition, window sizes larger than 14 increasingly overshadowed the fine-grain dynamics that we wanted to investigate. Thus we decided to use a window size of 14 to investigate the dynamics at the two week scale.

The second parameter to be chosen is a *window step size*. Window step size is how far the window moves up the line of synchrony before conducting CRQA again. We chose 1 for the step size to understand how the metrics evolve from day to day.

All other CRQA metrics remained the same as those previously noted.

**Fig 10:**
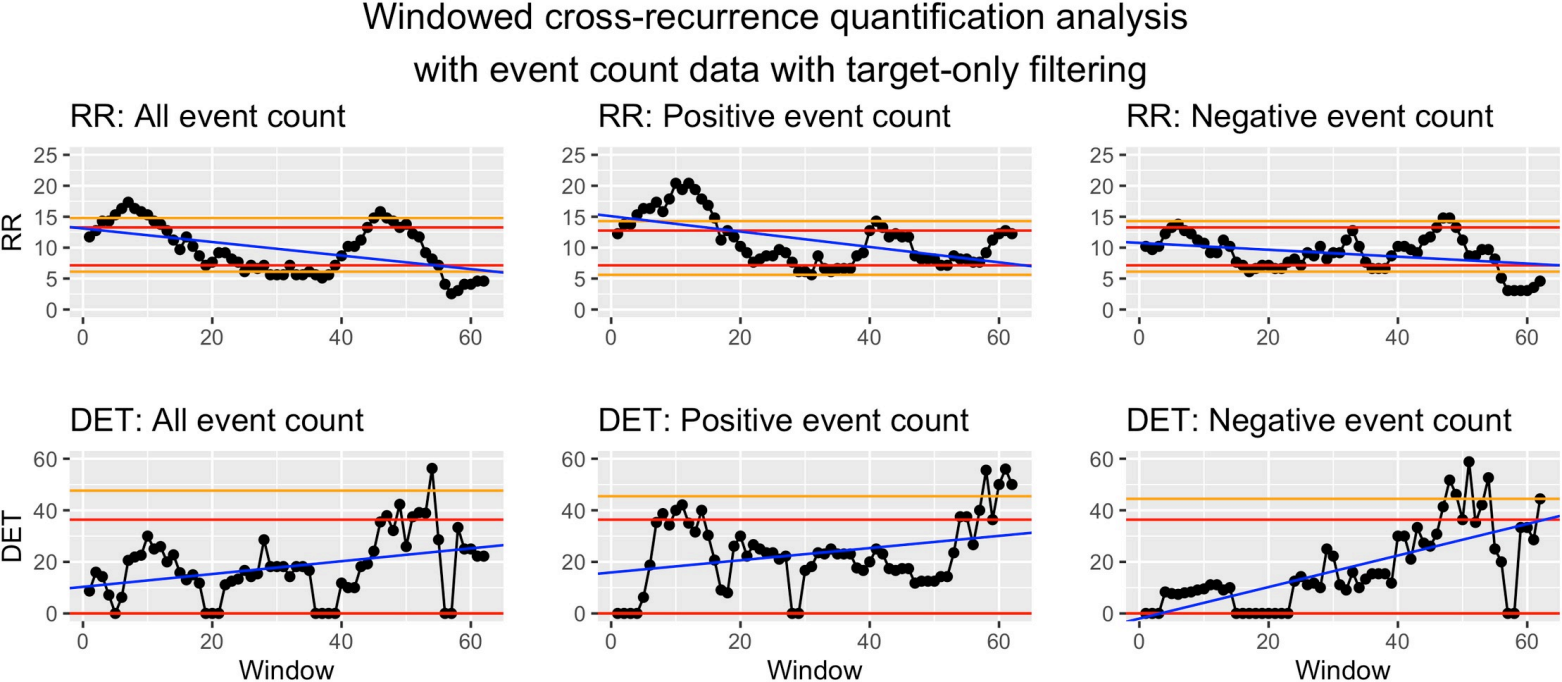
Windowed CRQA for social cohesion and event counts from target filtered data.

**Fig 11:**
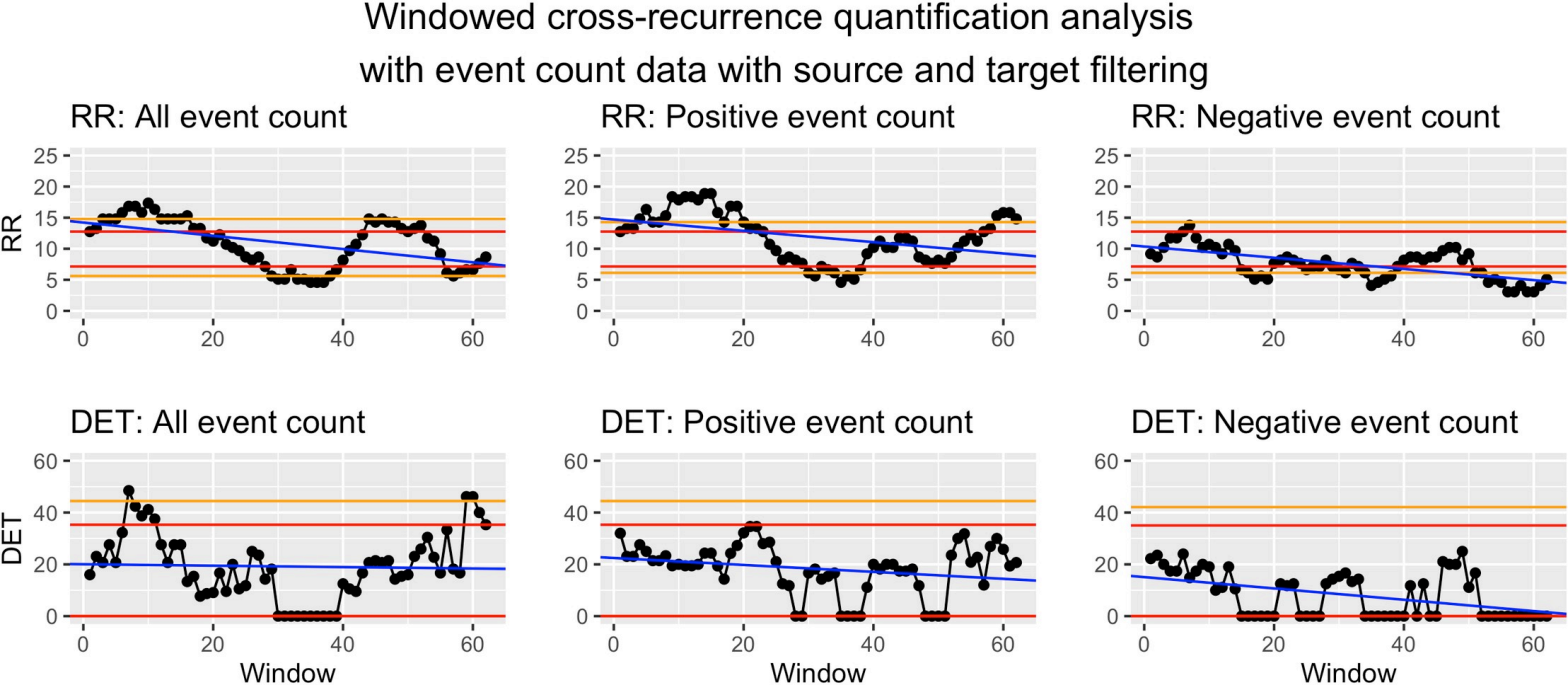
Windowed CRQA for social cohesion and event counts from source and target filtered data.

*Visualization*. To visualize WCRQA, the resulting metrics from each window can be plotted as a time series. This allows for visual interpretation of the pattern or fluctuation of the metrics across time. These dynamics can then be linked to other external events. See Figs 10 and 11 for examples.

*Approximate permutation test*. To visualize the differences in the WCRQA metrics from those obtained by chance, a permutation test was done. 1000 permuted time series were generated (without replacement) of length 14, (in accordance with the 14-day windows of real data for our WCRQA analyses), for each real time series; WCRQA was conducted on each combination of cohesion and event permuted time series. The upper and lower 95th and 99th percentiles for significance were set based on the distribution of the permuted WCRQA metrics.

## Results

### Cross-recurrence quantification analysis

#### Syria as target

A permutation test for the CRQA of social cohesion and count of all events found a statistically significant difference from chance for DET, NRLINE, and LAM. All other metrics did not reach significance (Fig 8; Table 1).

**Table 1:** CRQA results for target-only data.

metric	all events	p	sig.	positive events	p	sig.	negative events	p	sig.
DET	26.218	0.001	***	22.689	0.022	*	22.353	0.029	*
NRLINE	73.000	0.000	***	63.000	0.038	*	62.000	0.037	*
maxL	4.000	0.056	.	5.000	0.003	**	4.000	0.048	*
L	2.137	0.256		2.143	0.202		2.145	0.234	
ENTR	0.416	0.226		0.397	0.256		0.420	0.225	
rENTR	0.379	0.547		0.286	0.814		0.382	0.565	
LAM	30.756	0.015	*	28.403	0.034	*	27.059	0.052	.
TT	2.179	0.171		2.449	0.024	*	2.333	0.060	.

*p <* .*10*

* p < .*05*

** p < .*001*

A permutation test for the CRQA of social cohesion and count of positive events found a statistically significant difference from chance for DET, NRLINE, maxL, LAM, and TT. All other metrics did not reach significance (Fig 8; Table 1).

A permutation test for the CRQA of social cohesion and count of negative events found a statistically significant difference from chance for DET, NRLINE, and maxL. All other metrics did not reach significance (Fig 8; Table 1).

#### Syria as source and target

A permutation test for the CRQA of social cohesion and count of all events found a statistically significant difference from chance for DET, maxL, and LAM. All other metrics were non-significant (Fig 9; Table 2).

**Table 2:** CRQA results for source and target data.

metric	all events	p	sig.	positive events	p	sig.	negative events	p	sig.
DET	22.017	0.047	*	23.193	0.019	*	21.008	0.129	
NRLINE	61.000	0.068	.	60.000	0.111		59.000	0.126	
maxL	4.000	0.035	*	4.000	0.054	.	3.000	0.395	
L	2.148	0.196		2.300	0.000	***	2.119	0.374	
ENTR	0.425	0.193		0.687	0.001	***	0.364	0.382	
rENTR	0.387	0.499		0.626	0.034	*	0.525	0.128	
LAM	32.773	0.004	**	28.739	0.032	*	21.681	0.216	
TT	2.267	0.084	.	2.192	0.163		2.115	0.322	

*p <* .*10*

* p < .*05*

** p < .*001*

A permutation test for the CRQA of social cohesion and count of positive events found a statistically significant difference from chance for DET, L, ENTR, rENTR, and LAM (Fig 9; Table 2).

A permutation test for the CRQA of social cohesion and count of negative events found no statistically significant difference from chance for and metrics (Fig 9; Table 2).

### Windowed cross-recurrence quantification analysis

In the WCRQA plots, the 95th percentiles are represented by horizontal red lines, and the 99th percentiles are represented by horizontal orange lines. The trend lines are plotted in blue.

#### Syria as target

For social cohesion and count of all events, there was a consistent period of at or above-chance RR from the 3rd–12th and 44th–50th window. DET was largely above chance from the 47th to the 54th window, with some exceptions (Fig 10).

For social cohesion and count of positive events, we saw a downward trend in RR as the window advances throughout the sample. Interestingly, we see largely above-chance RR until the 16th window, with an exception for window 1. A peak also appeared for the 40th–42nd windows. DET was intermittently at or above chance for the 8th–14th and 54th–62nd windows (Fig 10).

For social cohesion and count of negative events, RR stayed above or almost above chance from the 5th–6th and 46th–49th windows and was below chance for an extended period from the 56th–62nd window. DET bottomed out at zero for the 15th–23rd windows. The metric was above or nearly above chance only toward the end of the time series, in the 47th–54th and 62nd windows (Fig 10).

#### Syria as source and target

For social cohesion and count of all events, there was a consistent period of above- or nearly above-chance RR in the first 23 windows and the 44th–52nd windows. RR was consistently below chance for the 29th–39th windows. DET showed above- or nearly above-chance values in windows 7–11 and 59–62 (Fig 11).

For social cohesion and count of positive events, there was a downward trend in RR as the window moved, with above- or nearly above-chance values in the first 23 windows and the 57th–62nd windows. RRR was largely below-chance for the 30th–38th windows. DET stayed largely within chance with intermittent bottoming-out to a value of 0 (Fig 11).

For social cohesion and negative events, RR was above chance for the 7th window and below chance for the 15th–19th windows, intermittently below chance between the 25th–39th windows, and below chance for the 51st–62nd windows. DET showed a downward trend as the windows moved, with no above-chance values. It bottomed out to zero for the 15th–20th and 24th-27th windows, intermittently bottomed out for the 34th–45th windows, and consistently bottomed out for the 52nd–62nd windows (Fig 11).

## Discussion

In the present study, we investigated the dynamics of real-world events and social cohesion on Twitter in Syria during Arab Spring. All analyses were also run with the deciled count of daily original tweets and deciled count of daily retweets to examine whether the results could be solely driven by the volume of Twitter activity. Details and results can be found in Appendix A. We found some significant connections between events and volume of original tweets and retweets, but these patterns of results differed in important ways from the pattern of results identified in the analyses of social cohesion. This suggests that—although tweet volume and social cohesion are not independent—tweet volume and social cohesion are conceptually distinct from one another. This is further supported by separate cross-correlation analyses between the social cohesion metric and original tweets and between cohesion and retweets (see Appendix A); in both analyses, relations between social cohesion and each of these metrics showed different patterns and did not reach statistical significance at lag 0 (i.e., simultaneity). We thank an anonymous reviewer for suggesting these complementary analyses. CRQA methodology allows us to disentangle the relationship between sociolinguistic cohesion and exogenous political events. This work stands alongside the model of Palestinian-Israeli social media and events developed by Zeitzoff [5] and the role of peripheral participants in the Syrian Arab Spring movement by Steinert-Threlkeld [4]. By introducing CRQA and WCRQA to political science as time series analysis methods, we demonstrate how language and social mobilization interact with events along the conflict-cooperation continuum in Syria. We have introduced a novel theoretical framework of sociolinguistic cohesion, which we operationalize using a weighted measure of lexical relationships among social media posts. Using nonlinear dynamical time series analyses, we investigated the theory that virtual mobilization is increasingly reflective of real-world events during times of strife.

### Cross-recurrence quantification analysis

#### Syria as target

The significant result of DET and NRLINE for social cohesion with all events supports our first hypothesis that event activity is coupled with online social cohesion. The frequency of all events are thus associated with a greater number and greater length of shared trajectories with social cohesion than would be expected by chance. This could be due to a conflation of the different kinds of events. However, when we specifically target positive events, the coupling with social cohesion is strong, running counter to our second hypothesis. In addition to the significant DET, NRLINE, and LAM metrics seen in the all-events analysis, maxL and TT are significant for positive events, suggesting a more stable relationship. This may be a reflection of citizens’ reactions to events that align with their cause. An act of concession by the government or aid from another country may have led to an increase in morale or feelings of hope, in turn leading to a groundswell of support on Twitter in an attempt to boost morale.

Negative event frequency also showed coupling with social cohesion, as indicated by significant DET, NRLINE, and maxL metrics. The significant result for maxL again suggests greater stability of coupling with negative events than with all events. Perhaps during times of intense strife, individuals in Syria focused on communication and public awareness, turning to Twitter to report and rally together. Further text analysis should be conducted to decipher the underlying tone of the tweets.

#### Syria as source and target

By adding Syria as a source in the data filtering, the event dataset increased in size by 1690 observations. These additional observations were times in which Syria initiated an event with another country. Events initiated by Syria but directed internally within the country were already captured by the target-only filtering. Taking outwardly directed events into account changed the coupling for all-event filtering methods. There was no longer a significant number of shared trajectories or line lengths for social cohesion and count of all events. The recurrent points were still more likely to fall in diagonal trajectories (as seen in the significant DET metric), but there were fewer trajectories overall. This indicates that the cohesion on Twitter is not as strongly tied to the frequency of overall daily events when considering Syria as the target country, as they are less likely to share the same intensity values across extended periods of time. Since Syria-as-source events must be at the root of this difference, it is plausible that Syrians were focused on the target events as they are immediately impacting them; integrating additional source events would have then overshadowed the relationship between target events and social cohesion online.

There was also a slight change in coupling between positive events and social cohesion that once again can only be due to the externally directed events. These two time series showed greater stability in their coupling (reflected in L) but lower attractor strength (reflected in maxL). This means that—although the longest trajectory in the target-only analysis was disrupted by the addition of source events—the additional observations resulted in an overall greater average length of trajectories. However, the significant ENTR and rENTR metrics suggest that there was more variability in the coupling. Rather than interpreting these two changes as a fundamental change in coupling between source events and positive events, this may indicate that the trajectories formed by the target events were broken by the additional source events. This would also explain the overall decrease in the number of shared trajectories (reflected in NRLINE).

The coupling between social cohesion with negative events changed dramatically. All previously significant metrics (DET, NRLINE, maxL) became non-significant. Similarly to that of positive events, this change may not be reflective of source events themselves but rather a disruption of the relationship with target events. The negative outward-directed source events may not have been of interest to Syrian Twitter users. Those external events may have then overshadowed the true coupled system between negative target events and virtual social cohesion.

While interesting, the underlying meaning or drivers of these changes in CRQA results requires further investigation that incorporates the actual content of the tweets themselves. One possible reason (among many) may be that the social cohesion of a population in the midst of an uprising are more tightly coupled to the state of the country itself, rather than the externally directed actions taken by the ruling class to manage international relations. For example, we observed coupling between negative events and social cohesion when considering only the target-filtered data, but that coupling disappeared when we added the source-filtered data to our dataset. In other words, the source events—that is, those taken by Syria toward parties outside of Syria (since Syrian-directed, Syrian-initiated actions were already included in the target-only dataset)—may have muddied the relationship between different kinds of target events and Twitter social cohesion—that is, the “singing with one voice” of the Syrian populace as they protested against their government. However, we cannot test this hypothesis with the current time series data; future research should explore the semantic content of this tweet corpus to determine the nature of the socially cohered language.

### Windowed-recurrence quantification analysis

**Fig 12:**
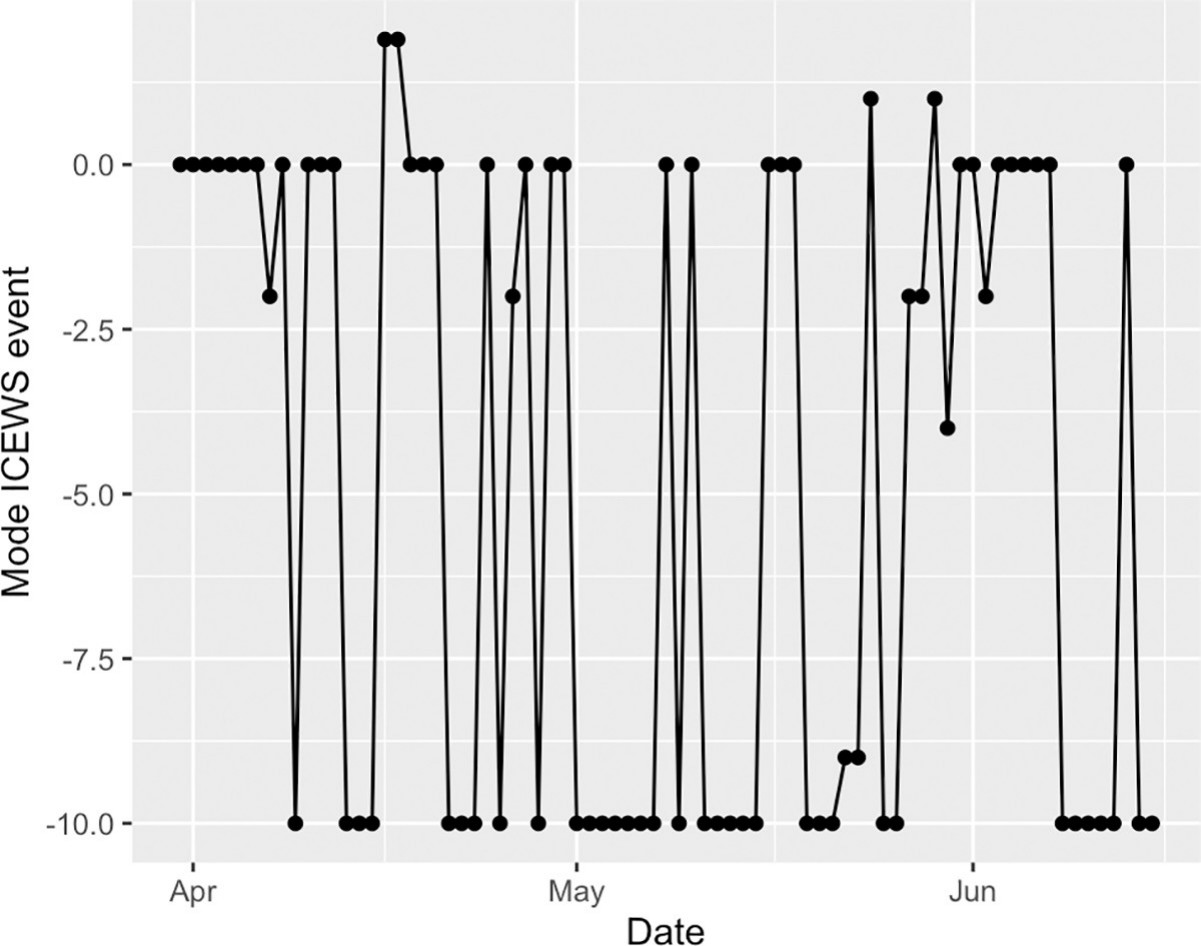
A plot of the most frequently documented ICEWS event score per day.

*Syria as target*. For the RR values in WCRQA of social cohesion and count of all events (Fig 10), we see both an early and late above-chance peak. However, DET for these periods only rises above chance for the later peak. During this later period, the modal ICEWS event for each day (Fig 12) was almost consistently -10, the most violent event category. This supports our first hypothesis, that overall online cohesion may reflect the frequency of events occurring on the ground, particularly those that are violent in nature.

For positive events, we see a similar early peak in RR, but the later above-chance values did not match up temporally with that of the all-events time series (Fig 10). This suggests that the early stretch of above-chance RR may have been captured in the RR of the all-events analysis, whereas the shorter stretches were overshadowed by the coupling with negative events. The early period of above-chance DET in the positive-event analysis aligned with the early period of above-chance RR, whereas the later stretch of significant DET values aligned temporally with that of the all-events analysis. It is possible that the later stretch reflected the decrease in the number of positive events happening (see Fig 4), while the relative sparsity of positive events led to an increase in coupling, with denizens rallying around dwindling bits of hope as time passed.

For negative events, RR rarely reached above-chance values, but in the later windows, there were above-chance peaks in both RR and DET that were similar to that of the all-events analysis. As mentioned above, this time period was almost consistently dominated by events with a score of -10, indicating the most violent, dramatic events. During this time, Twitter users may have come together to express grievances, bring about public awareness, ask for the help of other countries, or just document the events. However, further semantic analysis is needed to investigate these hypotheses.

The above-chance values for negative-event coupling are followed by a consistent period of below-chance RR and a drastic drop in DET, co-occurring with the increase in positive-event coupling. This again may reflect the shift in attention to some positive events (e.g., aid, ceasefire agreements) or even a decrease in hostilities. However, the present analyses should be complemented with future analyses of semantic content to investigate these possible explanations.

*Syria as source and target*. For count of all events and social cohesion, the WCRQA plot for RR looked largely the same as that of the target-only events, but DET showed a new early peak in above-chance values. This suggests that the overall number of events were more consistent from day to day when considering both source and target events, as compared to target-only events. In other words, days that previously had a more variable number of target events may now have had a more stable number of source and target events.

For positive events, we see a similar trend in RR as was observed in the target-only dataset, with a more pronounced late peak. However, the previously significant peaks in DET were no longer present. The addition of source events may have thus increased the amount of recurrence but overshadowed the trajectories of shared values found in the target-only events.

For negative events, the early peak in RR for the target-only filtering was dramatically reduced compared to the target-only dataset, now spanning only two windows. Similar to that of positive events, the late peak in DET was no longer present. We hypothesize again that the addition of source events may have obscured the true coupled relationship between the negative events and social cohesion.

The present work took a broad approach by explicitly including various ranges of event intensity. Future work will explore the relationship between events of intensely positive and intensely negative CAMEO scores (e.g., events with a score of 7 or higher and -7 or lower, respectively) and social cohesion to determine if the coupling strengthens with intensity. The decrease in coupling strength for negative events and social cohesion with the addition of source events observed in the present analyses could be due to an overall lower number of daily events; future analyses considering only high-intensity events would allow us to test this directly.

### Implications for methodology and theory

Nonlinear methods have been transformative for a variety of research areas (including psychology; [84]). One of the goals of the present work was to introduce variants of one kind of nonlinear method—specifically, recurrence quantification analysis—to this research area. While a number of excellent guides can provide detailed explanations of the methods and their statistical underpinnings (e.g., [69, 75, 85]), one of the most important considerations that should be briefly summarized here is about data.

Nonlinear methods—including recurrence-based analyses—require time series data. This requires repeated observations of the behavior of interest, preferably at a frequency that provides information about timing at a temporal resolution that allows the researcher to make observations about interesting variations over time. While this is easily controlled by scientists who conduct experimental research, it can be more difficult for scientists who rely on trace data or naturally occurring data. We recommend that political scientists work to expand efforts for improving the temporal granularity of event data where feasible. One possible means of achieving this may be through collaborative efforts like the Open Event Data Alliance [86].

After identifying the dataset of interest, researchers should think carefully about how they will winnow down their data for analysis. The differences in our findings when considering target-only or source-and-target filtering highlight the importance of considering directionality when using event data. The parties involved and the specific actions they take can dramatically influence results, and it is critical to think carefully about which will be chosen and why.

This methodology should be of great interest to interdisciplinary scientists investigating social mobilization. It has potential applications for gender studies and the #MeToo movement and for the relationship between social protest and repression during the Black Lives Matter protests. This methodological approach can help us better understand the fundamental human social mechanisms underlying social media and its influence on human behavior in real life. Given that many governments enact repressive measures when they determine that collective action by citizens is imminent, this methodology should help to improve the predictive power of using social media data in estimating repressive behavior. If social media messages are increasing in cohesion, this may represent a threat to civil and political liberties of denizens engaging in collective action, a unified reaction to sparse positive events, or an attempt to decry the government’s attempt to distract from what may really be going on.

## Conclusion

Considering the pervasiveness of social media, it is unsurprising that social activists and protesters have utilized these platforms to support real-world mobilization. Here, we quantitatively explored the connection between online social cohesion and real-world events. In addition to introducing political scientists to a family of powerful nonlinear dynamics analyses, our results have serious implications for monitoring global movements. While state-run media in regions of authoritarian rule are often biased and serve as propaganda for the leader and central government, the self-organized nature of Twitter provides a new avenue to gain insight into truths about uprisings and social mobilization. By conceptualizing Twitter and real-world events as two elements of an inextricably linked dynamical system, the levels of social cohesion within social media platforms could be monitored for fluctuations indicating shifts in power and peace.

## Appendix A

To investigate if the results presented in this article were solely due to volume of online activity, we ran CRQA and windowed CRQA with the time series of number of original tweets and retweets per day. All other parameters and processes remained the same as those reported in the Method section. For original tweets, the CRQA target-only and source-target results show differences in significant metrics for all events, positive events, and negative events (Tables 3 and 4). For retweets, the CRQA target-only and source-target results show differences in significant metrics for all events, positive events, and negative events (Tables 5 and 6).

**Table 3:** CRQA results for original tweets and target-only data.

metric	all events	p	sig.	positive events	p	sig.	negative events	p	sig.
DET	24.958	0.003	**	26.138	0.000	***	24.115	0.007	**
NRLINE	68.000	0.007	**	70.000	0.001	***	65.000	0.016	*
maxL	4.000	0.042	*	4.000	0.051	.	4.000	0.049	*
L	2.176	0.101		2.214	0.031	*	2.200	0.032	*
ENTR	0.491	0.089	.	0.552	0.022	*	0.535	0.025	*
rENTR	0.447	0.317		0.502	0.216		0.487	0.206	
LAM	31.535	0.009	**	28.499	0.030	*	26.813	0.053	.
TT	2.174	0.173		2.414	0.028	*	2.338	0.053	.

*p <* .*10*

* p < .*05*

** p < .*001*

**Table 4:** CRQA results for original tweets and source-target data.

metric	all events	p	sig.	positive events	p	sig.	negative events	p	sig.
DET	25.632	0.001	***	26.981	0.000	***	26.138	0.000	***
NRLINE	69.000	0.002	**	75.000	0.000	***	72.000	0.000	***
maxL	4.000	0.058	.	3.000	0.376		4.000	0.053	.
L	2.203	0.037	*	2.133	0.270		2.153	0.182	
ENTR	0.532	0.035	*	0.393	0.277		0.448	0.145	
rENTR	0.484	0.221		0.567	0.083	.	0.408	0.462	
LAM	32.378	0.004	**	28.162	0.042	*	22.091	0.190	
TT	2.259	0.093	.	2.197	0.159		2.113	0.327	

*p <* .*10*

* p < .*05*

** p < .*001*

**Table 5:** CRQA results for retweets and target-only data.

metric	all events	p	sig.	positive events	p	sig.	negative events	p	sig.
DET	27.150	0.000	***	24.958	0.001	***	22.934	0.028	*
NRLINE	74.000	0.000	***	64.000	0.027	*	61.000	0.064	.
maxL	4.000	0.038	*	5.000	0.007	**	4.000	0.045	*
L	2.176	0.077	.	2.312	0.000	***	2.230	0.015	*
ENTR	0.490	0.061	.	0.713	0.000	***	0.576	0.016	*
rENTR	0.446	0.313		0.514	0.163		0.524	0.174	
LAM	31.535	0.009	**	28.499	0.030	*	26.813	0.053	.
TT	2.174	0.173		2.414	0.028	*	2.338	0.053	.

*p <* .*10*

* p < .*05*

** p < .*001*

**Table 6:** CRQA results for retweets and source-target data.

metric	all events	p	sig.	positive events	p	sig.	negative events	p	sig.
DET	26.476	0.001	***	28.499	0.001	***	26.644	0.001	***
NRLINE	73.000	0.001	***	77.000	0.001	***	73.000	0.000	***
maxL	4.000	0.038	*	5.000	0.005	**	4.000	0.043	*
L	2.151	0.184		2.195	0.049	*	2.164	0.125	
ENTR	0.418	0.213		0.519	0.042	*	0.468	0.103	
rENTR	0.381	0.566		0.374	0.569		0.426	0.385	
LAM	32.378	0.004	**	28.162	0.042	*	22.091	0.190	
TT	2.259	0.093	.	2.197	0.159		2.113	0.327	

*p <* .*10*

* p < .*05*

** p < .*001*

**Fig 13:**
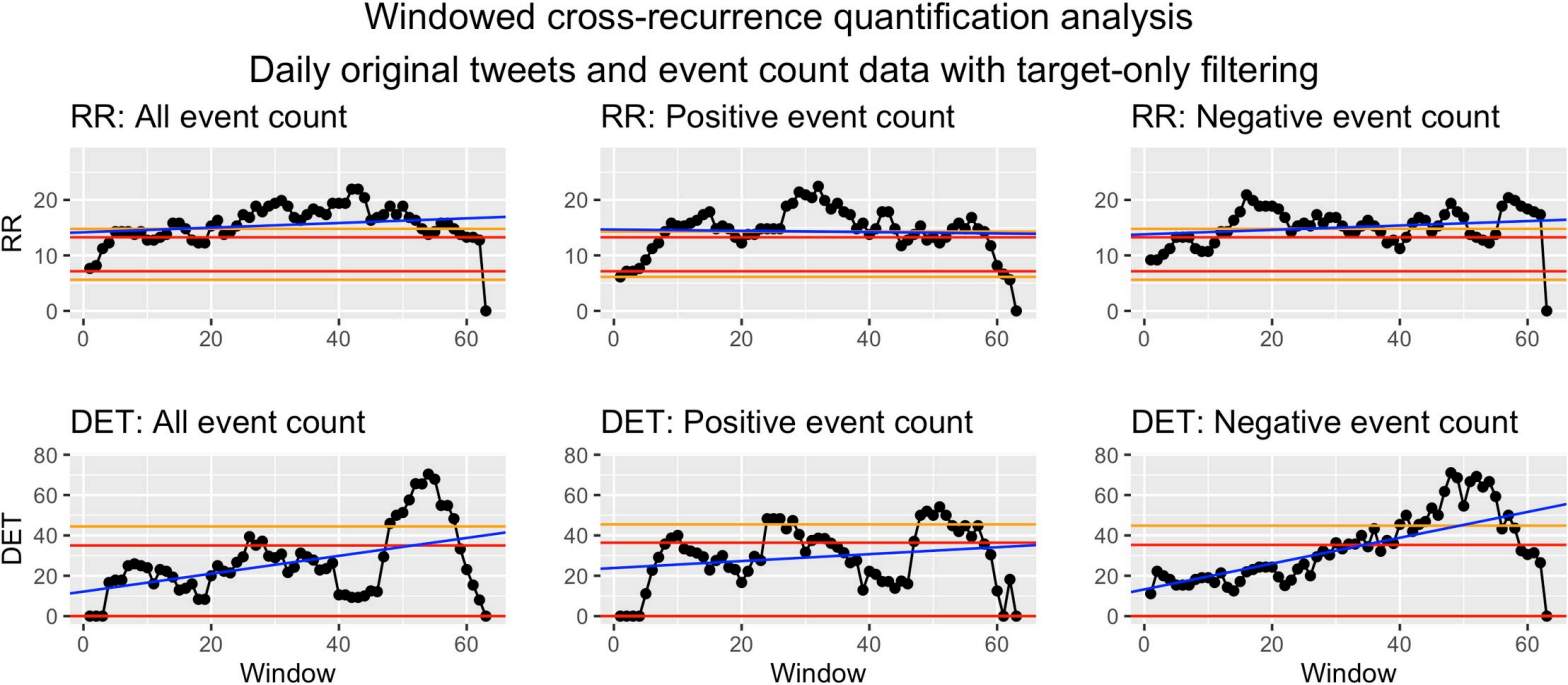
Windowed CRQA for daily original tweets and event counts from target filtered data.

**Fig 14:**
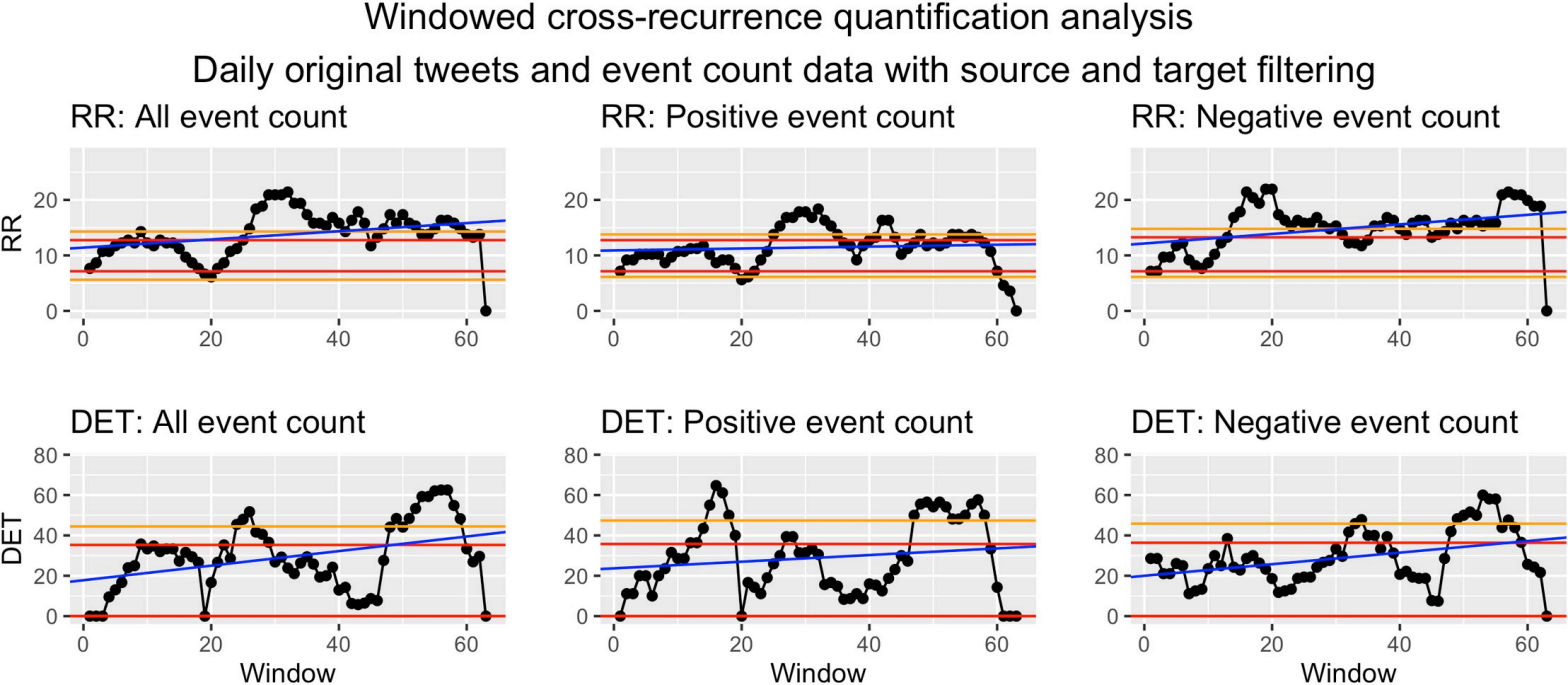
Windowed CRQA for daily original tweets and event counts from source-target filtered data.

**Fig 15:**
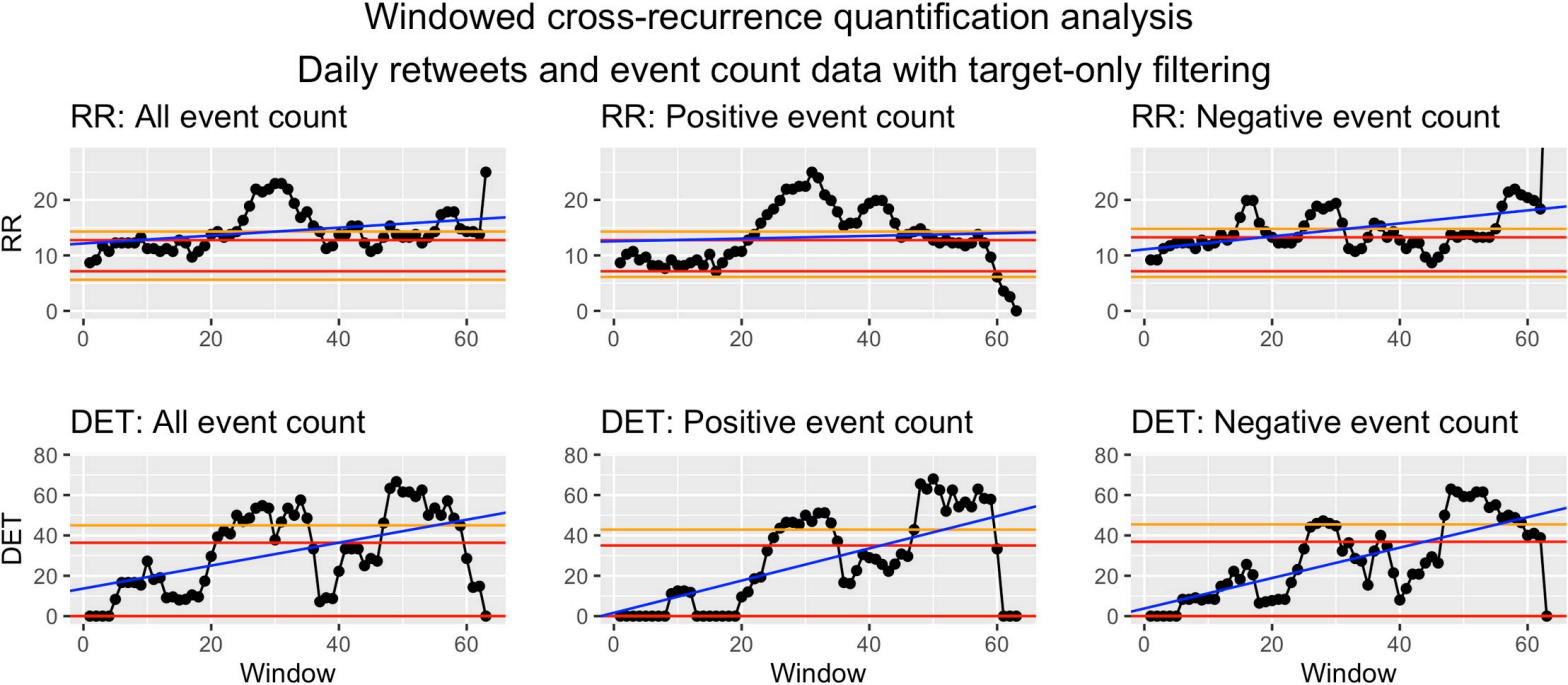
Windowed CRQA for daily retweets and event counts from target filtered data.

**Fig 16:**
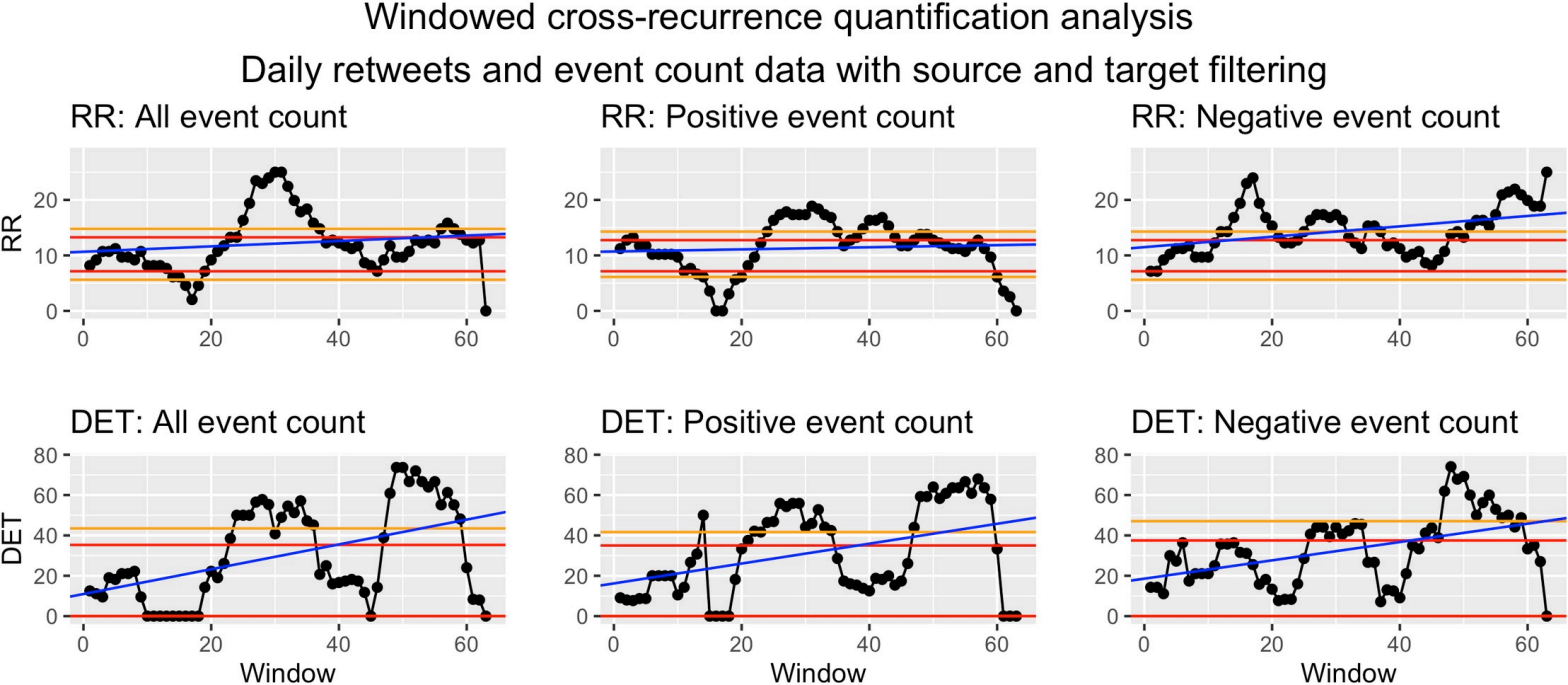
Windowed CRQA for daily retweets and event counts from source-target filtered data.

For windowed CRQA, the evolution of both RR and DET for the retweet and original tweet count time series show different patterns for both the target-only and source-target event analyses (Figs 13–16).

**Fig 17:**
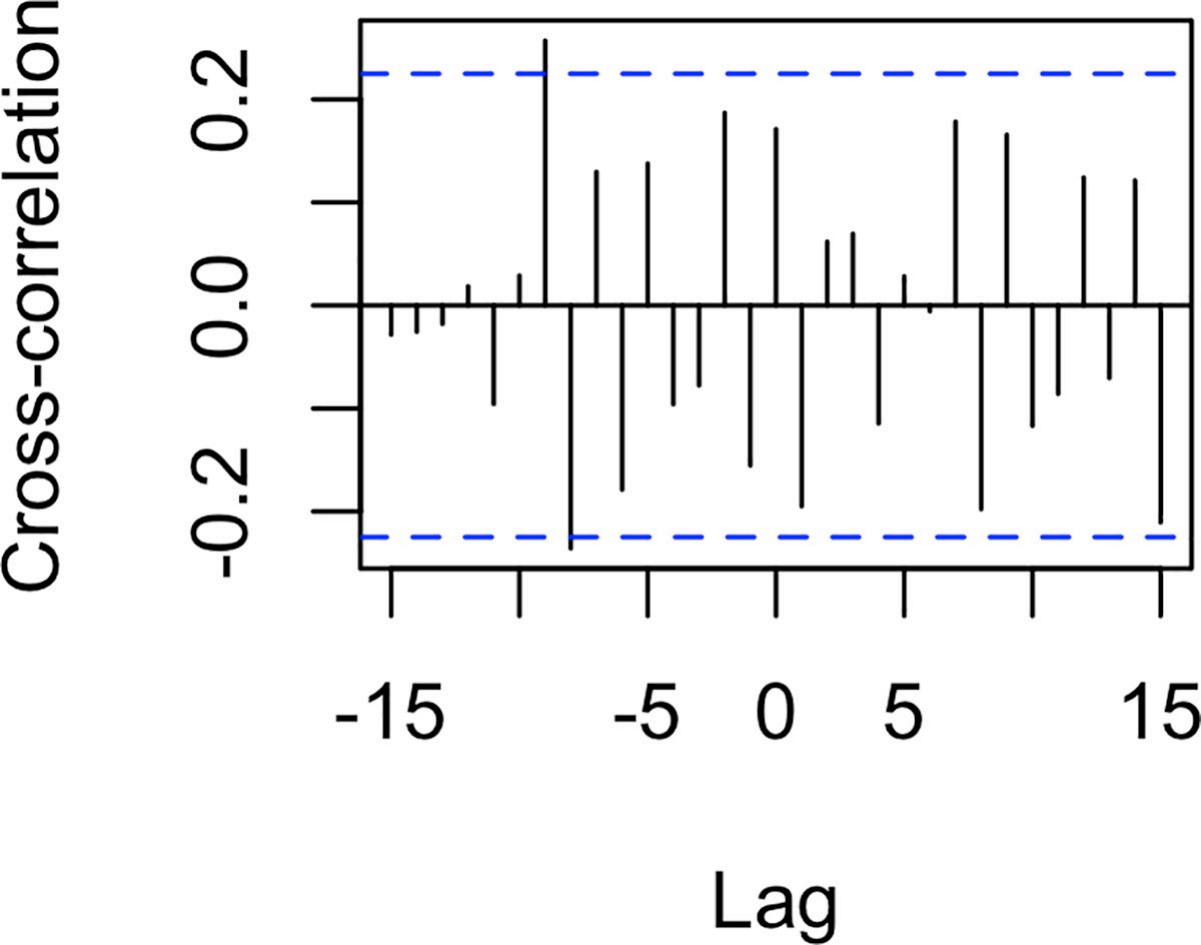
Cross-correlation plot for social cohesion and count of daily original tweets.

**Fig 18:**
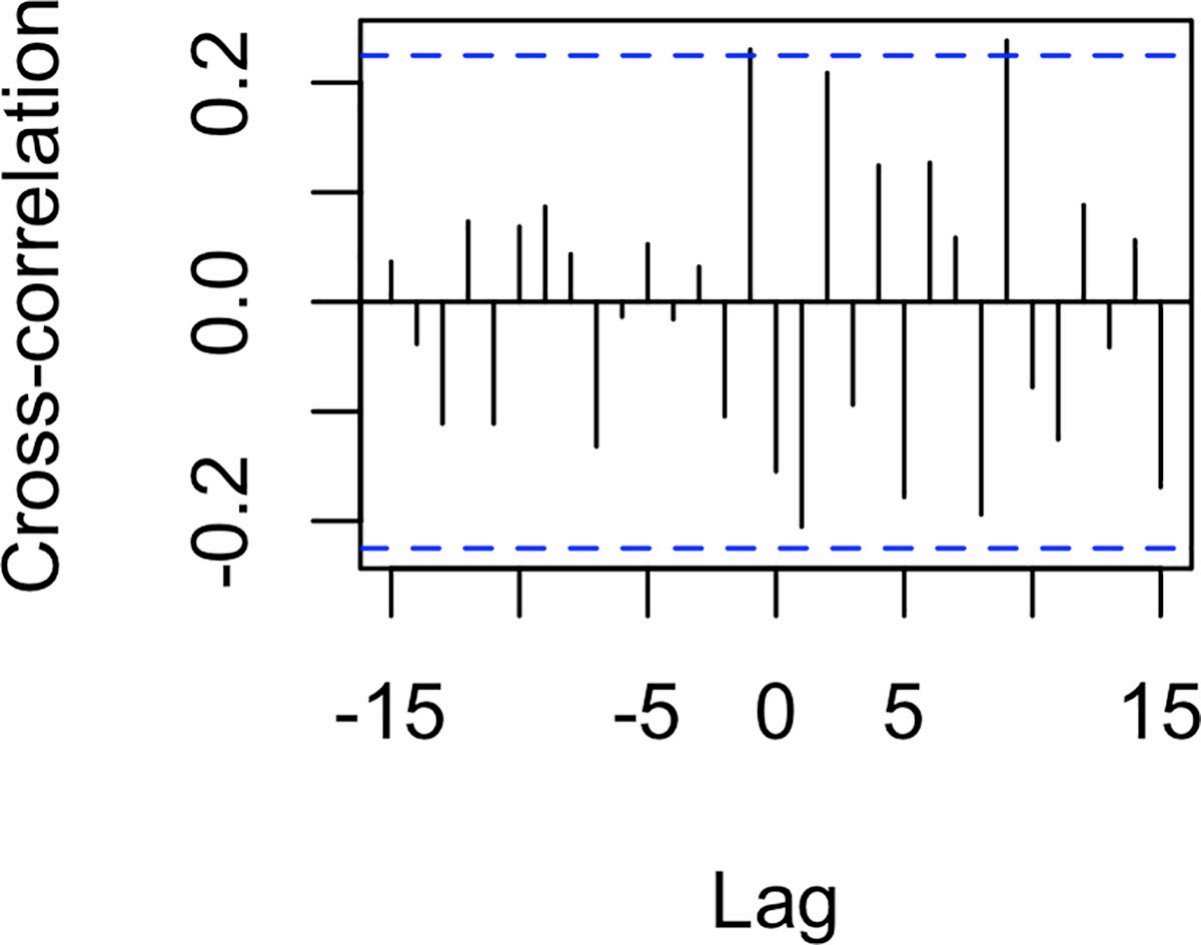
Cross-correlation plot for social cohesion and count of daily retweets.

Additionally, cross-correlation analyses between the social cohesion metric and original tweets and between cohesion and retweets showed different patterns and did not reach statistical significance at lag 0 (i.e., simultaneity; Figs 17 and 18).

The marked differences in results for both CRQA and WCRQA and the non-significant cross-correlation results at lag 0 for the time series of daily original tweets and daily retweets suggest strongly that the social cohesion metrics are not interchangeable with overall tweet or retweet volume. While we cannot say that the two are entirely independent from one another, social cohesion appears to be picking up patterns of linguistic cohesion among the tweets that differ in important ways from basic activity level of Twitter users.
